# Dual-mode hepatoprotective nanoplatform combines ROS scavenging and FGF21 activation for precision acute liver injury therapy

**DOI:** 10.1016/j.apsb.2026.03.005

**Published:** 2026-03-11

**Authors:** Xinyu Tan, Man Li, Zhibin Lan, Wenxiang Zhang, Junjie Zou, Yibo An, Jingwen Zheng, Xuwen Xiang, Jing Lin, Sheng Lin, Wenjie Ni, Lin Che, Gang Liu, Jing Chen, Zhixiang Lu

**Affiliations:** a Department of Child Health, Department of Pediatrics, Women and Children’s Hospital, School of Life Sciences, Xiamen University, Xiamen 361102, China; b State Key Laboratory of Vaccines for Infectious Diseases, Xiang An Biomedicine Laboratory, Fujian Provincial Key Laboratory of Innovative Drug Target Research, School of Pharmaceutical Sciences, Xiamen University, Xiamen 361102, China; c State Key Laboratory of Molecular Vaccinology and Molecular Diagnostics, State Key Laboratory of Vaccines for Infectious Diseases, Center for Molecular Imaging and Translational Medicine, Xiang An Biomedicine Laboratory, School of Public Health, Xiamen University, Xiamen 361102, China; d Fuzhou University Affiliated Provincial Hospital, School of Medicine, Fuzhou University, Fuzhou 350108, China; e State Key Laboratory of Oncology in South China, Sun Yat-sen University Cancer Center, Guangzhou National Laboratory, Guangzhou 510005, China; f Sichuan Research Institute of Xiamen University, Chengdu 610000, China

**Keywords:** Acute liver injury, Reactive oxygen species, Multidimensional therapeutic strategy, Anti-inflammatory, Diselenide-bridged mesoporous silica nanosystem, Silymarin, FGF21, Precise delivery

## Abstract

To address the intertwined pathologies of oxidative stress, immune dysregulation, and lipid metabolic disorders in acute liver injury (ALI), we engineered an innovative nanosystem: M2@SAM-F21. M2@SAM-F21 features an M2 macrophage membrane coating encapsulating diselenide-bridged mesoporous silica nanoparticles for silymarin and FGF21 plasmid DNA codelivery, enabling synergistic multidimensional treatment. First, reactive oxygen species (ROS)-responsive oxidative stress is mitigated. Diselenide bonds undergo ROS-triggered cleavage, achieving precise silymarin release that efficiently scavenges multiple ROS types, significantly mitigating hepatic oxidative damage. Simultaneously, FGF21 drives immune reprogramming. Efficiently expressed FGF21 protein drives macrophage polarization toward the M2 anti-inflammatory phenotype, effectively reducing proinflammatory cytokine levels, with TNF-*α* decreased to 61 pg/mL and IL-6 reduced to 20 pg/mL, while increasing IL-10 to 68 pg/mL to reverse the immune microenvironment imbalance. In addition, metabolic homeostasis is restored. Activated fatty acid oxidation pathways substantially decrease serum concentrations of total cholesterol (TC) to 4.984 mmol/L and triglycerides (TG) to 0.586 mmol/L, and almost reduce hepatocellular lipid droplet accumulation by 76%, resolving metabolic dysregulation. In both alcoholic and drug-induced ALI models, M2@SAM-F21 demonstrates exceptional hepatoprotection. For example, it significantly restores serum alanine aminotransferase (ALT) to 46.6 U/L and aminotransferase (AST) to 180 U/L in the alcoholic ALI model, while markedly alleviating histopathological vacuolization and steatosis, with biological distribution imaging confirming inflammation-targeted accumulation and proteomic analysis further elucidating therapeutic mechanisms. Exhibiting superior biosafety and dual therapeutic-prophylactic potential, this system serves as a pioneering paradigm that integrates targeted delivery, intelligent release, and multipathway synergy for ALI treatment.

## Graphical abstract

A biomimetic nanoplatform enables targeted, dual-mode therapy combining antioxidant defense and FGF21 activation to precisely treat acute liver injury.

**Figure:**
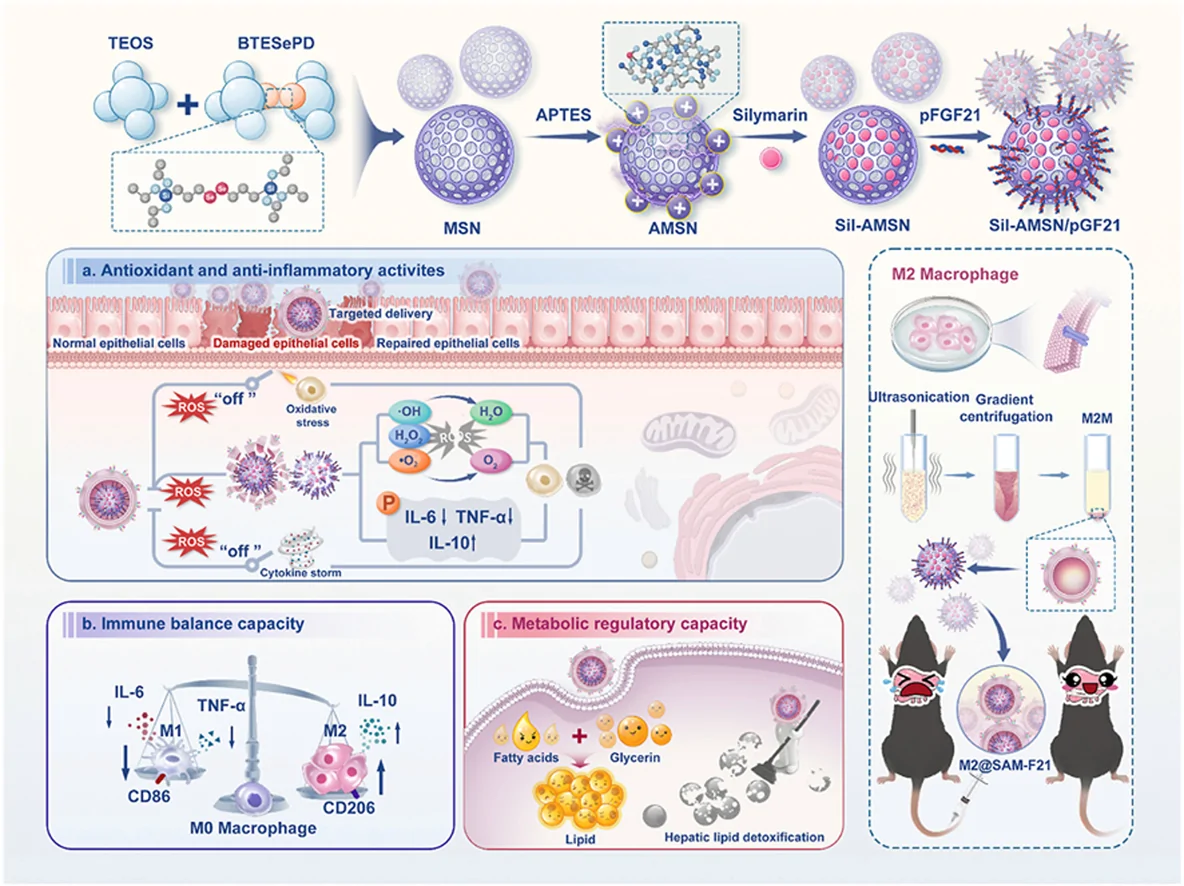

## 1 Introduction

As the largest visceral organ in the human body, the liver plays a central role in maintaining overall health and metabolic vitality^1^. Acute liver injury (ALI) is characterized by extensive hepatocyte damage occurring over a short period and clinically manifests as liver dysfunction and hepatocyte necrosis. Data from the World Health Organization indicate a rising incidence of ALI, driven by factors such as drug abuse, pesticide exposure, environmental pollution, and unhealthy lifestyle habits. ALI can be categorized into various types, including alcoholic, drug-induced, chemical, and viral forms, on the basis of etiology and mechanism^2^, ^3^, ^4^. Despite the diversity of triggers, a core common mechanism underlying ALI pathogenesis is the massive generation of reactive oxygen species (ROS)^5^. Excessive ROS triggers hepatocytes to release a surge of inflammatory cytokines (*e.g.*, TNF-*α* and IL-6) and apoptosis-related proteins, establishing a vicious cycle that exacerbates hepatocyte damage. This cascade may ultimately progress to chronic liver disease, cirrhosis, or even hepatocellular carcinoma^5^, ^6^, ^7^, ^8^. Regrettably, effective clinical strategies for preventing and treating ALI are limited.

Clinical consensus emphasizes the critical importance of the early identification of ALI and the correction of reversible causes, necessitating appropriate pharmacological intervention to prevent progression to liver failure. Hepatoprotective agents represent a commonly utilized class of clinical drugs, with mechanisms of action encompassing improvements in liver function, the enhancement of detoxification capacity, and the promotion of hepatocyte regeneration^9^. Among the diverse array of available hepatoprotective drugs, the antioxidant silymarin has been extensively studied. Substantial evidence has demonstrated that silymarin possesses significant anti-inflammatory, antioxidant, and antifibrotic properties and has diverse biological and pharmacological activities; it has shown potential efficacy across various domains through antiviral, anti-infective, antitumor, hepatoprotective, and lipid-lowering effects^10^^,^^11^. For example, a double-blind, prospective, randomized controlled trial conducted by Ferenci et al.^12^ involving 170 patients with cirrhosis demonstrated that silymarin treatment reduced mortality. Furthermore, Saller et al.^13^, through a systematic assessment of the clinical efficacy and safety of silymarin, concluded that at clinically achievable concentrations, silymarin exerts metabolic modulatory and cytoprotective effects. However, as a typical flavonolignan, silymarin has significant physicochemical limitations, such as extremely poor aqueous solubility and solubility in common organic solvents, resulting in low oral bioavailability. This key deficiency severely constrains its clinical application potential and its therapeutic efficacy. Overcoming this bottleneck requires the development of novel drug delivery systems to increase drug bioavailability and the establishment of optimized quality control protocols, representing urgent current priorities.

Fibroblast growth factor 21 (FGF21), an endocrine member of the fibroblast growth factor family, functions primarily as a hepatokine secreted by the liver in response to diverse metabolic stressors, including alcohol exposure and starvation^14^. Research has demonstrated the potential of FGF21 to mitigate ALI and fibrosis induced by iron overload, likely through the inhibition of iron overload-induced ferroptosis *via* the promotion of heme oxygenase-1 (HO-1) ubiquitination and the induction of nuclear factor erythroid 2-related factor 2 (Nrf2) expression^15^. Furthermore, Ye et al.^16^ investigated the pathophysiological role of FGF21 in APAP-ALI and proposed that markedly elevated FGF21 levels following APAP overdose may represent an endogenous compensatory mechanism. This mechanism counteracts drug-induced hepatotoxicity by augmenting antioxidant capacity in the liver through the peroxisome proliferator-activated receptor gamma coactivator 1-alpha (PGC-1*α*)/Nrf2 signaling pathway. However, the clinical translation of native FGF21 faces significant challenges: its susceptibility to proteolytic inactivation in plasma, short half-life, poor *in vivo* stability, and suboptimal biodistribution collectively limit its therapeutic potential^14^, ^15^, ^16^, ^17^. Gene therapy has emerged as a pivotal strategy in disease treatment, particularly for hepatic disorders, because the liver has been shown to uptake intravenously delivered nucleic acids (*e.g.*, DNA) with high efficiency^18^^,^^19^. Currently, gene delivery relies primarily on plasmid DNA (nonviral vectors) and viral vector systems; however, both remain constrained by a relatively limited gene cargo capacity and potential immunogenicity risks. Given the endogenous hepatoprotective role of FGF21, exploring strategies to upregulate its expression represents an attractive approach for synergistic ALI therapy.

Mesoporous silica nanoparticles (MSNs) have garnered significant attention in the field of drug delivery because of their high specific surface area, favorable biocompatibility, and tunable surface properties^20^. However, their relatively slow biodegradation kinetics raise concerns regarding potential long-term retention *in vivo* and associated risks of chronic toxicity^21^^,^^22^. Selenium (Se), a crucial trace element within the hepatic antioxidant defense system, plays a vital role in counteracting oxidative stress^23^. Notably, the bond energy of the diselenide bond (Se–Se, 172 kJ/mol) is significantly lower than that of the common disulfide bond (S‒S, 240 kJ/mol), indicating superior redox responsiveness^24^^,^^25^. Within microenvironments characterized by high levels of ROS, the diselenide bonds can undergo oxidative cleavage, effectively scavenging ROS^26^. Leveraging these properties, in this study, we innovatively integrated redox-sensitive diselenide bonds into the MSN framework, constructing a novel nanocarrier designed for the codelivery of silymarin and FGF21 plasmid DNA (pFGF21). Nevertheless, similar to all nanoparticle (NP)-based targeted delivery strategies, this platform faces inherent challenges. A primary obstacle is the rapid clearance of NPs by the immune system before they reach the target site, a critical limitation confronting nearly all nanodelivery systems^27^, ^28^, ^29^.

In recent years, cell membrane coating nanotechnology has emerged as a highly promising therapeutic platform^30^, ^31^, ^32^. This technique involves fusing naturally derived cell membranes onto the surface of synthetic NPs, enabling the NPs to inherit specific biological functionalities of the source cells, such as prolonged blood circulation and efficient active targeting to disease-related sites^33^^,^^34^. Macrophages, large and functionally versatile leukocytes, serve as central effector cells during inflammatory responses and tissue repair processes^35^^,^^36^. Previous studies have demonstrated that macrophage membrane-coated NPs exhibit high targeting efficiency and favorable therapeutic outcomes in various inflammatory disease models, including those of colitis^37^, cancer^38^, and liver injury^39^. Importantly, macrophages play a dual regulatory role in ALI, influencing both tissue destruction and resolution/repair events. Polarization toward a proinflammatory (M1) phenotype involves the secretion of proinflammatory cytokines and chemokines, the activation of intracellular cell death pathways, the destruction of adjacent tissue, the inhibition of cellular proliferation, and the exacerbation of inflammation^40^^,^^41^. Conversely, polarization toward an anti-inflammatory/pro-reparative (M2) phenotype involves the release of anti-inflammatory factors (*e.g.*, IL-10) and clearance of necrotic cellular debris, promoting inflammation resolution, sequential tissue repair, and cellular proliferation^42^^,^^43^. Consequently, the extent of liver inflammation and the progression of repair are largely governed by the dynamic interplay between the M1 and M2 macrophage polarization states. Modulating macrophage polarization is recognized as an effective strategy for mitigating ALI^44^. On the basis of this rationale, the M2 macrophage membrane (M2M) was used in this study to coat the aforementioned nanocarrier. The aim of this strategy was not only to increase carrier stability and minimize drug leakage through the physical membrane barrier but also, more importantly, to endow the carrier with M2 macrophage-associated biological functions, with the objective of reversing dysregulated immune homeostasis within the ALI microenvironment.

Building upon the aforementioned research background and strategy, in this study, we successfully constructed a biomimetic codelivery system coated with an M2 macrophage membrane (designated M2@SAM-F21). The core of this system comprises MSNs integrated with redox-sensitive Se‒Se bonds that are capable of simultaneously encapsulating silymarin and pFGF21. The aim of this design is to achieve targeted synergistic treatment for ALI (Scheme 1). The M2@SAM-F21 system possesses three key advantages: 1) enhanced stability and active targeting—the M2 membrane coating acts as a physical barrier, reducing premature drug leakage during systemic circulation. Furthermore, the enrichment of chemokine receptors on the membrane surface endows the system with a specific ability to actively target hepatic inflammatory sites; 2) two-pronged strategy for drug delivery—the elevated ROS microenvironment characteristic of ALI triggers the cleavage of diselenide bonds within the carrier framework while simultaneously activating its intrinsic antioxidative function; this intelligent responsive system enables the on-demand and precise release of silymarin at the pathological site; and 3) efficient gene delivery and endogenous protein expression—this system facilitates the efficient delivery of pFGF21 to hepatocytes, promoting the expression and secretion of the endogenous FGF21 protein. Ultimately, the system exerts its therapeutic effect through multitarget intervention mechanisms: (i) exogenous ROS clearance—direct scavenging of ROS by the exogenous antioxidant drug silymarin and ROS-responsive diselenide bonds (Se–Se), effectively blocking oxidative stress cascades; (ii) anti-inflammatory and endogenous regulation—activation of FGF21-mediated hepatoprotective pathways that regulate lipid metabolism and mitochondrial protection and suppress apoptotic signaling; and (iii) multidimensional synergy–coordinated mechanisms—antioxidant, anti-inflammatory, and metabolic regulation—significantly attenuate hepatocyte necrosis and inflammatory infiltration in ALI while facilitating tissue repair (Scheme 1). This work pioneers a novel dual-track therapeutic platform based on an “exogenous scavenging–endogenous activation” strategy, providing a new paradigm for precision intervention in ALI.

**Scheme 1:**
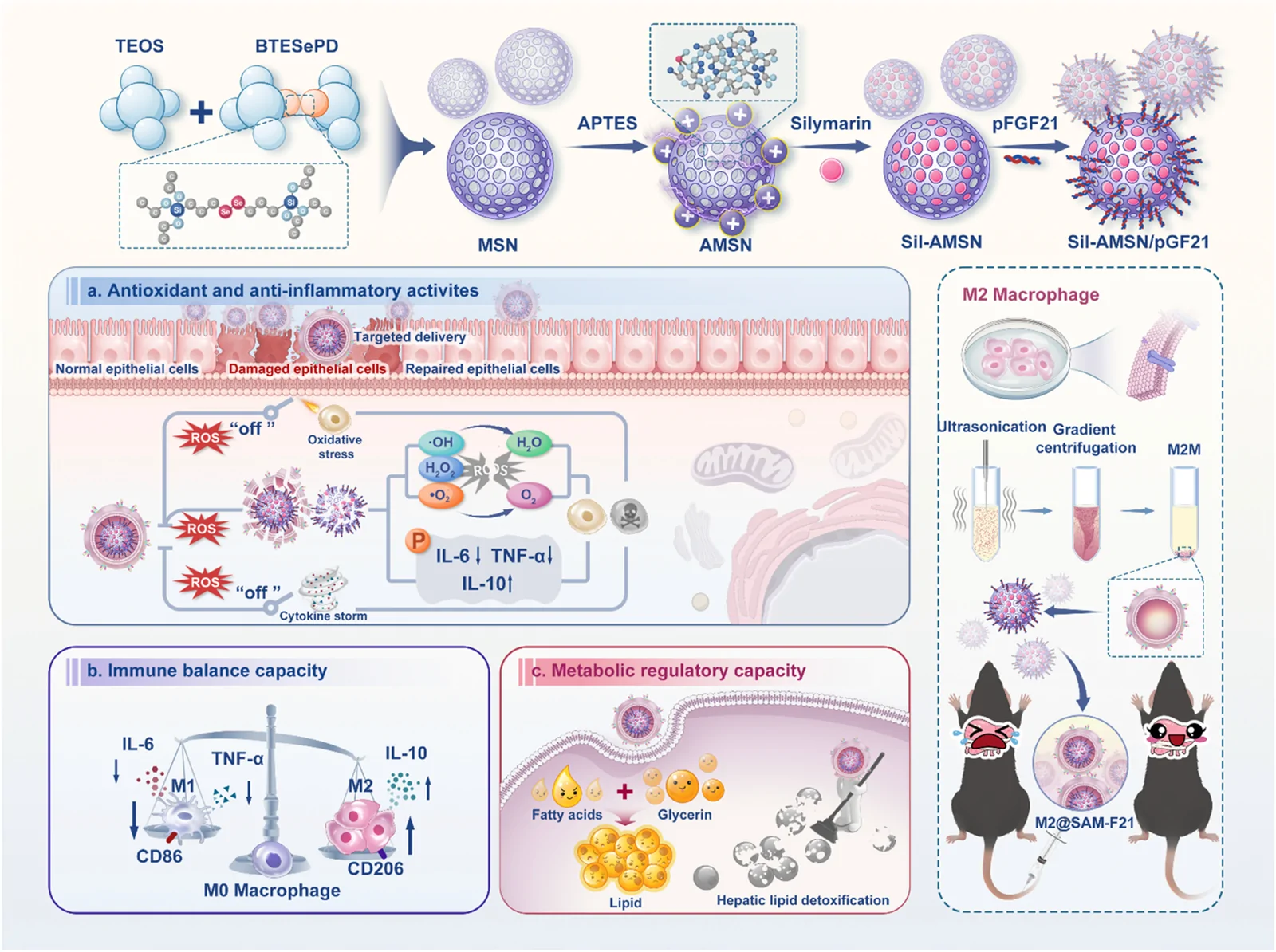
Schematic illustration of M2@SAM-F21 nano-therapeutic platform. The M2@SAM-F21 nano-therapeutic platform features a diselenide-bridged mesoporous silica core co-loaded with Silymarin and FGF21 plasmid, enveloped by an M2 macrophage membrane coating for inflammation-targeted delivery and immune evasion. This integrated system operates through triple synergistic mechanisms: (a) antioxidant and anti-inflammatory activities, (b) immune balance capacity, and (c) metabolic regulatory capacity. Those collectively enable synergistic suppression of oxidative stress storms, inflammatory cascades, and lipid dysregulation in acute liver injury (ALI).

## 2 Materials and methods

### 2.1 Materials

Silymarin (Sil) and acetaminophen (APAP) were obtained from TargetMol Chemicals Inc. (BOSTON, USA). (3-Aminopropyl) triethoxysilane (APTES) and tetraethyl orthosilicate (TEOS) were obtained from Adamas Reagents Co., Ltd. (Shanghai, China). (Bis(3-(triethoxysilyl)propyl) diselenide-silane was obtained from Xi’an ruixi Biological Technology Co., Ltd. (Xi’an, China). Triethanolamine (TEAH_3_) was obtained from RHAWN Reagents Co., Ltd. (Shanghai, China). Cetyl Trimethyl Ammonium p-Toluene Sulphonate (CTAT) was obtained from HEOWNS Co., Ltd. (Tianjin, China). Lieber-DeCarli Ethanol Rat Diet and Lieber-DeCarli Regular Control Diet were purchased from dyets Reagents Co., Ltd. (Wuxi, China). DPPH and potassium persulfate (K_2_S_2_O_8_) were purchased from Aladdin Biochemical Technology Co., Ltd. (Shanghai, China). ABTS was obtained from Shanghai Yuanye Bio-Technology Co., Ltd. (Shanghai, China). Dimethyl sulfoxide (DMSO) and *N*,*N*-dimethylformamide (DMF) were obtained from Chengdu Kelong Chemical Co., Ltd. (Chengdu, China). RS102895 hydrochloride was obtained from Macklin Technology. DCFH-DA (2′,7′-dichlorodihydrofluorescein diacetate), Calcein-AM and propidium iodide (PI) were bought from Yeasen Biotechnology Co., Ltd. (Shanghai, China). Dulbecco’s modified Eagle’s medium (DMEM)/F12, Dulbecco’s modified Eagle’s (DMEM) medium, Fetal bovine serum (FBS) and penicillin/streptomycin were provided by Thermo Fisher Bio-chemical Product (Beijing) Co., Ltd. (Beijing, China). Phosphate Buffered Saline (PBS, 0.01 mol/L, pH 5.5–7.4) was obtained from Wuhan Servicebio Technology Co., Ltd. (Wuhan, China). 4% paraformaldehyde was acquired from Wuhan Servicebio Technology Co., Ltd. (Wuhan, China).

### 2.2 Instrumentation

TEM images of MSN, Sil-AMSN, and M2@SAM-F21 were obtained by using a transmission electron microscope (JEOL 2100F). The ultraviolet (UV)–Vis spectrum was acquired using an Agilent spectrophotometer. The FT-IR spectra were acquired by FT-IR spectrophotometer (IR Affinity-1S). Mapping analysis was carried out on an X-ray energy-dispersive spectrometer (Bruker XFlash 5030T, Germany). X-ray photoelectron spectroscopy (XPS, Thermo Fisher Scientific K-Alpha, USA) was used for surface elemental analysis. The bio-distribution of M2@SAM-F21-ICG was characterized by fluorescence small animal live imaging system (Carestream FX Pro, Carestream). The correlation indexes of mouse ALT, AST, TC, and TG were analyzed by automatic biochemical analyzer (Mindray, BS-240Vet). Thermo Scientific plate reader was used to measure cell viability results. All the measurements were performed at room temperature if not specially mentioned.

### 2.3 Cells

AML-12 cells were obtained from Immocell Co., Ltd. (Xiamen, China) and they were maintained in DMEM/F-12 supplemented with 10% FBS and 1% penicillin–streptomycin. Raw 264.7 macrophages (ATCC, TIB-71) were cultured in DMEM containing 10% FBS and 1% penicillin–streptomycin. Both cell lines were incubated at 37 °C in a 5% CO_2_ humidified atmosphere.

### 2.4 Animals

All the animal experiments were authorized by the Institutional Research Ethics Committee of Xiamen University (Approved number: XMULAC20210102). Male C57BL/6J mice (6–8 weeks) were obtained from Xiamen University Laboratory Animal Center (Xiamen, China). All animal experiments were conducted in compliance with the Guidelines for the Care and Use of Laboratory Animals of Xiamen University. All parameters related to animal welfare and experimental endpoints, including body weight, clinical scores, and behavioral assessments, were recorded prior to the induction of euthanasia. To maintain consistency and reduce variability, all animals were euthanized using an identical protocol (agent, dose, operator) across experimental groups, and tissues were harvested within a standardized time window after death confirmation.

### 2.5 Construction of the FGF-21-expressing plasmid

The plasmid pFGF21 was purchased from Hunan Fenghui Biotechnology Co., Ltd.

### 2.6 Synthesis of M2@SAM-F21

Synthesis of MSN: First, we prepared diselenide-bridged mesoporous silica nanoparticles (MSN) *via* the sol–gel method. We dissolved 0.06 g of cationic template CTAT and 0.015 g of catalyst TEAH_3_ (Triethanolamine) in 10 mL of deionized water, stirring at 80 °C for 30 min to form a micellar solution. Then, we pre-mixed 0.4 g of silica precursor TEOS (tetraethyl orthosilicate) and 0.1 g of diselenide precursor BTESePD (Bis(3-(triethoxysilyl) propyl) diselenide-silane) in 1 mL of ethanol. This mixture was slowly added dropwise to the micellar solution and stirred vigorously at 80 °C at 1000 rpm for 4 h for co-hydrolysis and condensation. After the reaction, the product was collected by centrifugation, washed three times with ethanol to remove surfactants, and refluxed with 1% ammonium nitrate ethanol solution for 12 h to completely remove the template. Finally, the resulting grey–white solid was vacuum-dried to obtain the functionalized MSN carrier. Its uniform selenium distribution and mesoporous structure laid the foundation for subsequent drug loading.

Synthesis of AMSN: Amine-functionalized mesoporous silica nanoparticles (designated AMSN) were prepared *via* two distinct approaches using 3-aminopropyltriethoxysilane (APTES): For the co-condensation method, 0.067 μL APTES was introduced into the reaction mixture 20 min after TEOS addition to enable simultaneous hydrolysis and amine incorporation; alternatively, the post-grafting method involved dispersing 10 mg pre-formed MSNs in 7 mL ethanol under sonication, followed by sequential addition of 300 μL deionized water and 150 μL glacial acetic acid with 10-min stirring to activate surface silanols, after which 100 μL APTES was dropwise added and reacted for 24 h under continuous stirring. Nanoparticles from both methods were purified through three cycles of ethanol/water washing and centrifugation, yielding AMSN for subsequent applications.

The loading of silymarin: Dissolve 10 mg of AMSN and 5 mg of silymarin in 2 mL of DMF at room temperature in the dark on a stirrer at 1000 rpm for 24 h.

The loading of pFGF21: In a 4 °C environment, incubate 10 μg of pFGF21 with varying amounts of AMSN or Sil-AMSN (100, 200, 400, and 500 μg), formed at mass ratios of 0, 10:1, 20:1, 40:1, and 50:1 (μg/μg) for 30 min.

Extraction of M2 macrophage membranes: Confluent M2 macrophages were harvested using cell scrapers into ice-cold PBS (2 mL per 100 mm dish), followed by three freeze-thaw cycles (stored at −80 °C for 4–8 h with intermittent thawing at room temperature.) to initiate cellular disruption. Protease inhibitor PMSF was added (1:100 *v*/*v*) before continuous vortex lysis at 4 °C for 30 min. The lysate underwent pulsed sonication on ice (10 s pulses × 10 cycles at 19% amplitude) for complete fragmentation. Sequential differential centrifugation was then performed: 2000 rpm (10 min, 4 °C) → 4000 rpm (10 min, 4 °C) → 5000 rpm (10 min, 4 °C) to remove nuclei and large debris. The supernatant was ultracentrifuged at 15,000×*g* (2 h, 4 °C) to pellet membrane fragments. Finally, the membrane vesicles were resuspended in 1 mL PBS, quantified *via* BCA protein assay (with protein concentration representing vesicle concentration), aliquoted, and stored at −80 °C.

M2 macrophage membranes coating: The Sil-AMSN/pFGF21 nanoparticles and isolated M2 macrophage membranes (M2M) were co-extruded through a 400 nm polycarbonate membrane using a mini-extruder at a 2:1 mass ratio (nanoparticles: membranes) for 20 consecutive passes. The resulting suspension was centrifuged at 12,000 rpm (10 min, 4 °C) to remove unbound membrane components, with the pelleted product designated as M2@SAM-F21. The coating efficiency of the M2 macrophage membrane was quantitatively determined using the BCA protein assay (Lablead, B5001).

### 2.7 Drug loading capacity

The encapsulation efficiency (*EE*%) of silymarin was determined *via* indirect UV spectrophotometry. First, a standard calibration curve of silymarin in DMF was established at 288 nm, demonstrating linearity (*R*^2^ > 0.99) over 0–80 μg/mL. Post-loading, nanoparticle suspensions were centrifuged at 15,000×*g* for 20 min. The supernatant absorbance was measured to calculate unbound drug concentration against the standard curve. Encapsulation efficiency was calculated according to Eq. (1):

(1) *EE*% = [(*W*_total_ – *W*_supr_)/*W*_total_] × 100

where *W*_total_ is total drug input, *W*_s_ᵤ_p_ᵣ is unbound drug in supernatant.

### 2.8 *In vitro* drug release kinetics

Drug release profiles were evaluated using a dynamic dialysis method. Precisely weighed M2@SAM-F21 nanoparticles were dispersed in 20 mL PBS release medium containing 2% Tween 80, simulating pathological (pH 5.5), physiological (pH 7.4), oxidative (100 μmol/L H₂O₂), and antioxidant (100 μmol/L H₂O₂ + VC) environments, respectively. Samples were incubated at 37 °C. At predetermined intervals (0.5, 1, 2, 4, 12, 18, 24, 36, and 48 h), 0.8 mL aliquots were withdrawn and replaced with equal volumes of pre-warmed fresh medium to maintain sink conditions. Withdrawn samples were filtered through 0.22 μm membranes, and silymarin concentrations were quantified against a pre-established UV standard curve (*λ* = 288 nm). Cumulative release percentage was calculated according to Eq. (2):

(2) Cumulative Release (%) = [(*C*_n_ × *V* + Σ*C*ᵢ × *V*_s_)/*M*_to_] × 100

where *C*_n_ is drug concentration at time *n*, *V* is total release medium volume (20 mL), *V*_s_ is sample volume withdrawn (0.8 mL), Σ*C*ᵢ is sum of previous sample concentrations, and *M*_tot_ is total drug loading.

### 2.9 Agarose gel electrophoresis assay

The binding characteristics of MSN and Sil-AMSN to pFGF21 plasmid were analyzed using a gel retardation assay: pDNA (fixed at 10 μg) was mixed with nanoparticles at varying mass ratios (0, 10:1, 20:1, 40:1, 50:1) in PBS and incubated for 30 min at room temperature to form complexes. After centrifugation (12,000×*g*, 10 min), supernatants were loaded onto 0.7% agarose gels containing 0.5 μg/mL GoldView nucleic acid stain. Electrophoresis was performed in 1 × TAE buffer at 150 V for 30 min. Unbound DNA bands were visualized using a Bio-Rad gel documentation system (Hercules, CA), with binding efficiency semi-quantitatively analyzed based on DNA band intensity.

### 2.10 Radical scavenging assays

To assess the antioxidant properties of M2@SAM-F21, ABTS·^+^ and DPPH· radical scavenging assays were performed.

ABTS‧^+^ scavenging activity Assay: ABTS radical cation (ABTS‧^+^) stock solution was generated by mixing 7.4 mmol/L ABTS and 2.6 mmol/L potassium persulfate in equal volumes, followed by 12-h dark incubation. The resulting solution was diluted with acetate buffer (pH 4.5) to achieve an absorbance of 0.70 ± 0.02 at 734 nm. Test samples (MSN or M2@SAM-F21) at concentrations ranging from 0 to 200 μg/mL were mixed with the diluted ABTS‧^+^ solution (typically 1:10 *v*/*v* sample to oxidant ratio) and incubated under dark conditions at 25 °C for 30 min. Absorbance was measured at 734 nm against a blank. Radical scavenging activity was calculated according to Eq. (3):

(3) Scavenging (%) = [(*A*_c_ – *A*_s_)/*A*_c_] × 100

where *A*_c_ is absorbance of ABTS·^+^ control (no nanoparticles), and *A*_s_ is absorbance with nanoparticle treatment.

DPPH· scavenging activity Assay: A 0.1 mmol/L DPPH· radical solution was prepared in absolute ethanol with 2-h dark incubation under continuous stirring. Test samples (MSN or M2@SAM-F21 at 0–200 μg/mL) were combined 1:1 (*v*/*v*) with the DPPH· solution and reacted at 25 °C in the dark for 30 min. After post-incubation, the mixtures were centrifuged at 1500×*g* for 5 min to remove particulate interference. Absorbance of supernatants was measured at 517 nm against an ethanol blank. Radical scavenging capacity was calculated according to Eq. (4):

(4) Scavenging (%) = [(*A*_c_ – *A*_s_)/*A*_c_] × 100

where is *A*_c_ is Absorbance of DPPH· control (no nanoparticles), and *A*_s_ is Absorbance of sample-treated DPPH· solution.

### 2.11 H_2_O_2_ scavenging activity of M2@SAM-F21

H_2_O_2_ scavenging activity was determined using a commercial assay kit (Hydrogen Peroxide Assay Kit, A064-1-1, Nanjing Jiancheng Bioengineering Institute, China) following manufacturer protocols. M2@SAM-F21 was tested across a concentration gradient (0–200 μg/mL) for dose-dependent evaluation.

#### 2.11.1 O_2_·^-^ scavenging activity of M2@SAM-F21

O_2_·^-^ scavenging capacity was quantified using a commercial Superoxide Anion Assay Kit (A052-1-1, Nanjing Jiancheng Bioengineering Institute, China) according to manufacturer protocols. M2@SAM-F21 nanoparticles at graded concentrations (0–200 μg/mL) were introduced to the reaction system. Following 10-min incubation at ambient temperature, absorbance at 550 nm was measured using a microplate reader.

#### 2.11.2 ·OH scavenging activity of M2@SAM-F21

Reaction mixtures containing 1 mL of 6 mmol/L FeSO_4_, 1 mL of 6 mmol/L salicylic acid, and 1 mL of gradient-concentration M2@SAM-F21 (0–200 μg/mL) were initiated by adding 1 mL of 6 mmol/L H_2_O_2_. Following 30-min incubation at 37 °C, absorbance was measured at 510 nm. Scavenging efficiency was calculated according to Eq. (5):

(5) Scavenging (%) = [(*A*_0_ – *A*)/*A*_0_] × 100

where *A*_0_ is control absorbance without nanoparticles, and *A* is sample absorbance, whereby reduced absorbance directly corresponds to enhanced hydroxyl radical scavenging capacity.

### 2.12 *In vitro* biocompatibility evaluation of M2@SAM-F21

The cytotoxicity profiles of MSN, Sil-AMSN, and M2@SAM-F21 nanoparticles were evaluated using the CCK-8 assay. AML-12 hepatocytes and RAW 264.7 macrophages were seeded in 96-well plates at 1 × 10^4^ cells/well and pre-cultured for 24 h (37 °C, 5% CO_2_) to ensure adhesion. Following media aspiration, fresh medium containing nanoparticle suspensions (0–200 μg/mL) was administered. After 24 h exposure, cells were gently washed with PBS and incubated with 100 μL fresh medium containing 10 μL CCK-8 reagent (TargetMol, C0005) for 2 h at 37 °C. Absorbance at 450 nm was measured using a Varioskan Flash microplate reader (Thermo Scientific), with cell viability calculated relative to untreated controls according to Eq. (6), Complies with ISO 10993-5 cytotoxicity testing standards.

(6) Viability (%) = *A*_test_/*A*_control_ × 100

where *A*_test_ and *A*_control:_ represent absorbance of test and control wells, respectively. Triplicate wells per concentration required.

### *2.13* *In vitro* ROS scavenging ability of M2@SAM-F21

AML-12 cells (5 × 10^4^ cells/well) were seeded in 12-well plates for 24 h adherence. Following pretreatment with 40 μg/mL MSN, Sil-AMSN, or M2@SAM-F21 for 12 h, oxidative stress was induced by 10 mmol/L APAP exposure (4 h). ROS levels were quantified through parallel methodologies:

#### 2.13.1 Flow cytometry analysis

Trypsinized cells were centrifuged (300×*g*, 5 min), washed twice with ice-cold PBS, and incubated with 10 μmol/L DCFH-DA (2′,7′-dichlorodihydrofluorescein diacetate) in serum-free medium (30 min, 37 °C, dark). After PBS washing, DCF fluorescence (*E*_x_ = 488 nm, *E*_m_ = 525 nm) was immediately analyzed by flow cytometry (BD FACSVerse).

#### 2.13.2 Fluorescence microscopy

PBS-washed cells were loaded with 10 μmol/L DCFH-DA (30 min, 37 °C), rinsed thrice with serum-free medium, and imaged using a Leica DMi8 inverted microscope (DCF channel: 488 nm excitation, 500–550 nm emission).

### 2.14 Apoptosis detection

Apoptotic cells were quantified using an Annexin V-FITC/PI detection kit (BD Pharmingen™). Post-treatment cells underwent two ice-cold PBS washes, followed by EDTA-free trypsinization. Mechanically detached cells were centrifuged (300×*g*, 5 min), resuspended in 195 μL binding buffer, and stained with 5 μL Annexin V-FITC plus 10 μL propidium iodide (PI). After 15-min dark incubation on ice, samples were immediately analyzed by flow cytometry using standardized optical configurations: FITC detection at 488 nm excitation/530 nm emission, PI at 488 nm excitation/610 nm emission.

### 2.15 Live/dead assay of cells

Cell viability and death were quantified using Calcein-AM/PI dual fluorescence staining. After PBS washing to remove culture medium residues, cells were incubated with a freshly prepared staining solution (5 μL Calcein-AM + 15 μL PI in 5 mL serum-free medium) for 20 min in the dark. Post-incubation, three PBS washes removed excess dye. Fluorescence imaging was performed on a Leica DMi8 inverted microscope using standardized optical filters:

Live cells: Calcein-AM green fluorescence (*E*_x_ = 494 nm, *E*_m._ = 517 nm);
Dead cells: PI red fluorescence (*E*_x_ = 535 nm, *E*_m._ = 617 nm).

### 2.16 Measurement of cytokine secretion by ELISA

AML-12 hepatocytes were seeded in 6-well plates (3 × 10^5^ cells/well) and cultured for 24 h. Cells were pretreated with 40 μg/mL MSN, Sil-AMSN, or M2@SAM-F21 for 12 h, followed by 10 mmol/L APAP challenge for 4 h. Culture supernatants were collected and analyzed for inflammatory cytokines (TNF-*α*, IL-6 and IL-10) using species-specific ELISA kits, purchased from Neobioscience Technology Co., Ltd.

### 2.17 Mitochondrial membrane potential (MMP) test

MMP was evaluated using a JC-1 detection kit (Beyotime, C2003S). Cells pretreated with 50 μg/mL MSN, Sil-AMSN, or M2@SAM-F21 for 12 h were challenged with 10 mmol/L APAP for 4 h. Post-stimulation, cells underwent three PBS washes and incubation with JC-1 staining solution (37 °C, 5% CO_2_, 20 min). After serum-free medium washes and fresh medium equilibration, dual-channel fluorescence imaging was performed on a Leica DMi8 microscope with the following configurations:

JC-1 monomers green fluorescence (*E*_x_ = 488 nm, *E*_m_. = 525 nm);
JC-1 aggregates red fluorescence (*E*_x_ = 550 nm, *E*_m_. = 600 nm).

### 2.18 Immunohistochemistry

Begin by rinsing cells twice with PBS to remove residual culture medium. Fix cells with 4% paraformaldehyde (PFA) in PBS for 10 min at room temperature. After fixation, wash cells three times with PBS to eliminate fixative. Permeabilize cells using 0.1% Triton X-100 in PBS for 15 min at room temperature. Subsequently, block non-specific binding by incubating cells in 5% bovine serum albumin (BSA)/PBS blocking solution for 60 min at room temperature. Incubate cells with primary antibody diluted in blocking solution overnight at 4 °C. Following primary incubation, wash cells three times with PBS to remove unbound antibodies. Apply fluorophore-conjugated secondary antibody diluted in blocking solution for 45 min at room temperature (protected from light). Wash cells three times with PBS to clear excess secondary antibody. Finally, mount cells using DAPI-containing mounting medium for nuclear counterstaining and image with a fluorescence microscope (Zeiss LSM980).

### 2.19 Lipid droplet staining

#### 2.19.1 Cell culture

After co-treating AML-12 cells with 200 mmol/L ethanol, 40 μg/mL MSN, Sil-AMSN, and M2@SAM-F21 for 24 h, gently aspirate the culture medium and wash once with PBS.

#### 2.19.2 Oil Red O staining

Fix cells with 4% PFA for 10 min at room temperature, followed by two PBS washes. Add sufficient staining wash buffer to cover cells for 20 s. Aspirate and immediately apply freshly prepared Oil Red O working solution (Immocell, IMC-903) for 10–20 min incubation protected from light. Remove staining solution, add staining wash solution for 30 s, then aspirate. Rinse rapidly with PBS (20 s). Finally, cover cells with PBS and image lipid droplets using an optical microscope (Olympus).

#### 2.19.3 Bodipy 493/503 staining

At the designated timepoint, prepare 2 μmol/L BODIPY 493/503 staining solution in PBS. Rapidly rinse cells with 3 mL PBS to remove serum components. Premix BODIPY (TargetMol, T36957) solution with Hoechst (1:1000) and incubate with cells for 15 min at 37 °C in complete darkness. Post-staining, wash cells three times with PBS. Add sufficient PBS to cover cells and immediately image using a confocal microscope (Zeiss LSM980).

Microscope Configuration:

Hoechst, blue fluorescence (*E*_x_ = 405 nm, *E*_m_. = 480 nm);
BODIPY, green fluorescence (*E*_x_ = 488 nm, *E*_m_. = 550 nm).

#### 2.19.4 Nile Red staining

Using the Lipid Fluorescent Staining Kit (Nile Red Method, Solarbio, N8440-100), the working solution was prepared freshly according to the protocol. Premix Nile Red solution with Hoechst 33342 (1:1000) and incubate with cells for 15 min at 37 °C in complete darkness. Post-staining, wash cells three times with PBS. Add sufficient PBS to cover cells and immediately image using a confocal microscope (Zeiss LSM980).

### 2.20 Evaluation of biocompatibility

To assess the *in vivo* biocompatibility of M2@SAM-F21, male C57BL/6J mice (6–8 weeks, 20–25 g) received a single intravenous dose of M2@SAM-F21 (40 mg/kg), while control mice were administered an equivalent volume of saline. Blood sampling was performed on Days 1 and 7 post-injections for complete blood cell (CBC) analysis and serum biochemical assays, including liver function enzymes aspartate aminotransferase (AST) and alanine aminotransferase (ALT). Major organs (heart, liver, spleen, lungs, and kidneys) were subsequently harvested for histopathological examination through hematoxylin and eosin (H&E) staining.

### 2.21 Hemolysis assay

Fresh whole blood from healthy C57BL/6J mice was centrifuged (300×*g*, 15 min) to isolate erythrocytes. The erythrocytes were washed three times with PBS to remove plasma contaminants, then resuspended in PBS to achieve a homogeneous suspension. A 100 μL aliquot of this suspension was mixed with M2@SAM-F21 solutions at 1:10 (*v*/*v*) ratio and incubated at 37 °C for 3 h. Post-incubation, samples were centrifuged (13,000×*g*, 15 min) and supernatant absorbance measured at 540 nm using a spectrophotometer/microplate reader. Hemolysis ratio was calculated relative to controls: deionized water (100% hemolysis positive control) and PBS (0% hemolysis negative control).

### 2.22 Biodistribution

To evaluate the *in vivo* distribution of M2@SAM-F21, indocyanine green (ICG)-labeled nanoparticles (M2@SAM-F21-ICG) were administered to C57BL/6J mice with acetaminophen-induced acute liver injury (300 mg/kg APAP i.p. after 12 h fasting). Fifteen acute liver injury (ALI)-mice were randomized into three groups receiving intravenous injections (5 mg/kg ICG-equivalent dose) of: 1) free ICG, 2) Sil-AMSN-ICG, or 3) M2@SAM-F21-ICG. Whole-body fluorescence imaging was performed at 0.5, 2, 6, 12, and 24 h post-injection using the VISQUE InVivo Smart-LF system. At 24 h, mice were euthanized for *Ex vivo* fluorescence imaging of excised organs (heart, liver, spleen, lungs, and kidneys).

### 2.23 ALI model establishment

Male C57BL/6J mice (6–8 weeks, 20–25 g) were fasted for 12 h with ad libitum water access and administered a single intraperitoneal injection of APAP (300 mg/kg, TargetMol) to establish APAP-ALI, concurrently, EtOH-ALI was induced using the Lieber-DeCarli method involving: 5-day adaptation with 0–5% ethanol solid diet, 10-day maintenance on 5% ethanol liquid diet, and terminal gavage with 50% food-grade ethanol (5 g/kg).

### 2.24 Treatment and prevention of ALI in mice

Mice received intravenous injections (5 mg/kg) of MSN, Sil-AMSN, or M2@SAM-F21 with model-specific schedules: for the APAP-induced ALI, nanoparticles were administered within 2 h post-APAP injection; for the EtOH-induced ALI, daily treatments commenced 8 h post-gavage and continued for 7 days. Control groups received equivalent saline volumes. Terminal procedures were performed at 24 h (APAP-ALI) or 9 h after the final injection (EtOH-ALI), with serum and liver tissue collected for hepatic function assays and histopathological analysis.

#### 2.24.1 Measurement of ALT, AST, triglycerides (TG), and total cholesterol (TC)

Mice were euthanized for blood collection, and serum levels of ALT, AST, TG and TC were quantified using an automated biochemical analyzer.

#### 2.24.2 Malondialdehyde (MDA), total-superoxide dismutase (T-SOD) and glutathione (GSH) test

Mouse liver tissues were analyzed for MDA, T-SOD, and GSH levels using commercial assay kits: MDA (Solarbio, BC0025), T-SOD (Beyotime, S0101S), and GSH (Solarbio, BC1175), following manufacturers’ protocols. Tissue lysates were incubated with kit-specific working solutions for prescribed durations. Absorbances were measured *via* microplate reader, with concentrations calculated against standard curves.

#### 2.24.3 Evaluation of anti-apoptosis *in vivo*

Apoptosis in ALI model liver tissues was assessed by TUNEL assay. Frozen sections were fixed in 4% PFA (RT, 20 min), permeabilized with proteinase K (25 °C, 10 min), and labeled per manufacturer’s instructions. TUNEL-positive cells (red fluorescence) were quantified across six random fields per sample.

#### 2.24.4 Detection of superoxide production

Superoxide production was quantified in 5-μm frozen liver sections using dihydroethidium (DHE) staining. Sections were PBS-washed to remove cryoprotectant, then incubated with 1 mmol/L DHE (TargetMol, T2021) at 37 °C for 30 min in darkness. Upon superoxide-mediated oxidation, DHE converts to DNA-intercalating ethidium that emits red fluorescence. Post-incubation, sections underwent three 5-min PBS washes, followed by DAPI nuclear counterstaining. Fluorescent images were acquired on a confocal microscope (Leica DMi8), with DHE signal intensity indicating superoxide generation.

### 2.25 Western blot

Cells/tissues were lysed in RIPA buffer containing protease/phosphatase inhibitors (30 min, ice). Lysates were centrifuged (12,000×*g*, 10 min, 4 °C) and supernatants quantified using BCA assay (Beyotime, P0009). Equal protein aliquots (20–40 μg) were denatured in 5 × loading buffer (95 °C, 5 min), separated by 10% SDS-PAGE (120 V, 1.5–2 h), and transferred to PVDF membranes (300 mA, 2 h). Membranes were blocked with 5% non-fat milk/TBST (RT, 1 h) before overnight incubation with primary antibodies (4 °C in 5% BSA/TBST). After three 5-min washes with TBST, membranes were incubated with HRP-conjugated secondary antibodies (1:5000–1:10,000, 1 h, RT). Protein bands were visualized *via* ECL (Bio-Rad) using a ChemiDoc system, with band intensities quantified in ImageJ and normalized to GAPDH or *β*-actin.

Primary antibodies used were: CD86 (ABclonal, #A21198), CD206 (ABclonal, #A26948), FGF21 (Bioworld, #BS40478), SOD2 (Proteintech, #6647-1-Ig), HO-1 (Selleck, #A2413), Cd36 (Proteintech, #66395-1-Ig), ACOX1 (Proteintech, # 68017-1-Ig), HK2 (Selleck, #F0228), PKM2 (Selleck, #F0295), Arg1 (Abcam, #ab124917), Na^+^/k^+^-ATPase (ABclonal, #A11683), GAPDH (ABclonal, #A19056), Tubulin (ABclonal, #A12289), and *β*-actin (ABclonal, #AC026).

### 2.26 Immunocytochemistry

Deparaffinize and rehydrate sections, then fix with 4% PFA/PBS (10 min, RT). After 3 × 5 min PBS washes, permeabilize tissues with 0.1% Triton X-100/PBS (15 min, RT). Block non-specific sites using 5% BSA/PBS (60 min, RT), followed by primary antibody incubation in blocking solution (overnight, 4 °C). Remove unbound antibodies *via* 3 × 5 min PBS washes, then incubate with fluorophore-conjugated secondary antibody in blocking solution (45 min, RT; light-protected). After additional 3 × 5 min PBS washes, mount sections with DAPI-containing medium and image using a confocal microscope (Zeiss LSM980).

### 2.27 qRT-PCR

Total RNA was extracted from different liver samples using RNA isolater Total RNA Extraction Reagent (Vazyme Biotech Co., Ltd.). Subsequently, cDNA was synthesized with HiScript IV All-in-One Ultra RT SuperMix for qPCR (Vazyme Biotech Co., Ltd.). Quantitative PCR (qPCR) was then performed using SupRealQ Ultra Hunter SYBR qPCR Master Mix(U+) (Vazyme Biotech Co., Ltd.) by QuantStudio 3 Real-Time PCR System (Thermo Fisher Scientific) to measure target gene expression. Gene expression levels were normalized and are presented as fold changes relative to normal control samples.

### 2.28 Statistical analysis

Quantitative data is displayed as mean ± standard deviation (SD). Statistical differences were analyzed using a student’s *t*-test for two groups or one-way analysis of variance (ANOVA) for three or more groups. Statistical analyses were performed using GraphPad Prism 7 software. *P* values < 0.05 were considered statistically significant and illustrated by ∗*P* < 0.05, ∗∗*P* < 0.01, ∗∗∗*P* < 0.001, ∗∗∗∗*P* < 0.0001, and ns, no significance, respectively.

## 3 Results

### 3.1 Synthesis and characterization of M2@SAM-F21

A schematic diagram of the construction of M2@SAM-F21 is shown in Fig. 1A, the MSN core was fabricated through a sol–gel process, where diselenide bridges were introduced *via* oxidative coupling of selenol-containing silanes^45^, ^46^, ^47^. Transmission electron microscopy (TEM) (Fig. 1B) characterization revealed monodisperse spherical particles (diameter ≈70 nm), and dynamic light scattering (DLS) and elemental analysis (Supporting Information Fig. S1 and Fig. 1C) confirmed a uniform particle size distribution and homogeneous selenium dispersion. Given the negative surface charge of MSNs (−22.55 ± 1.46 mV), amino functionalization (yielding AMSNs) (Supporting Information Fig. S2) was performed by post-grafting modification to increase the loading capacity for negatively charged therapeutics (silymarin and pFGF21). This modification markedly reversed the zeta potential to 43.47 ± 1.11 mV (Supporting Information Fig. S3). Concurrently, Fourier transform infrared (FT-IR) (Fig. 1D) spectroscopy detected characteristic peaks at 3390 cm^−1^ (N–H stretching vibration) and 1650 cm^−1^ (N–H bending vibration), confirming successful amination, alongside peaks at 736 cm^−1^ (C–Se) and 563 cm^−1^ (Se–Se), verifying effective diselenide bond incorporation within the silica framework. Also, XPS spectra (Supporting Information Fig. S4) directly corroborates the conclusion drawn from the FT-IR results regarding the successful formation and preservation of the diselenide bridge during the synthesis and coating processes. Drug loading analysis (Supporting Information Figs. S5 and S6) demonstrated a silymarin loading efficiency of 31.57 ± 1.91% in the AMSNs. Furthermore, pFGF21 loading increased in a dose-dependent manner as the AMSN concentration increased from 100 to 500 μg (Fig. 1E). Notably, preloading with silymarin did not significantly impair the subsequent nucleic acid loading efficiency (Supporting Information Fig. S7). To construct the biomimetic delivery system, RAW264.7 cells were first polarized to the M2 phenotype *via* co-stimulation with IL-4 and IL-13 (Fig. 1F). Flow cytometry confirmed significantly higher CD206 expression in stimulated cells than in control cells (Fig. 1G). The M2 macrophage membranes were subsequently isolated through cell lysis and differential centrifugation and then used to coat the drug-loaded AMSNs, forming the M2@SAM-F21 composite (Supporting Information Figs. S8 and S9), as confirmed by Western blot analysis of characteristic M2 membrane markers and phosphorus-element mapping (Fig. 1H and Supporting Information Fig. S10). To validate the membrane coating efficiency, we employed the BCA protein assay. The membrane protein concentration extracted from 1 × 10^9^ cells was measured to be 527.917 μg/mL. The coating efficiency reached approximately 80% when the membrane extract was initially mixed with nanoparticles at a 1:1 volume ratio; further optimization by increasing the membrane-to-nanoparticle ratio enabled complete coating coverage.

**Figure 1:**
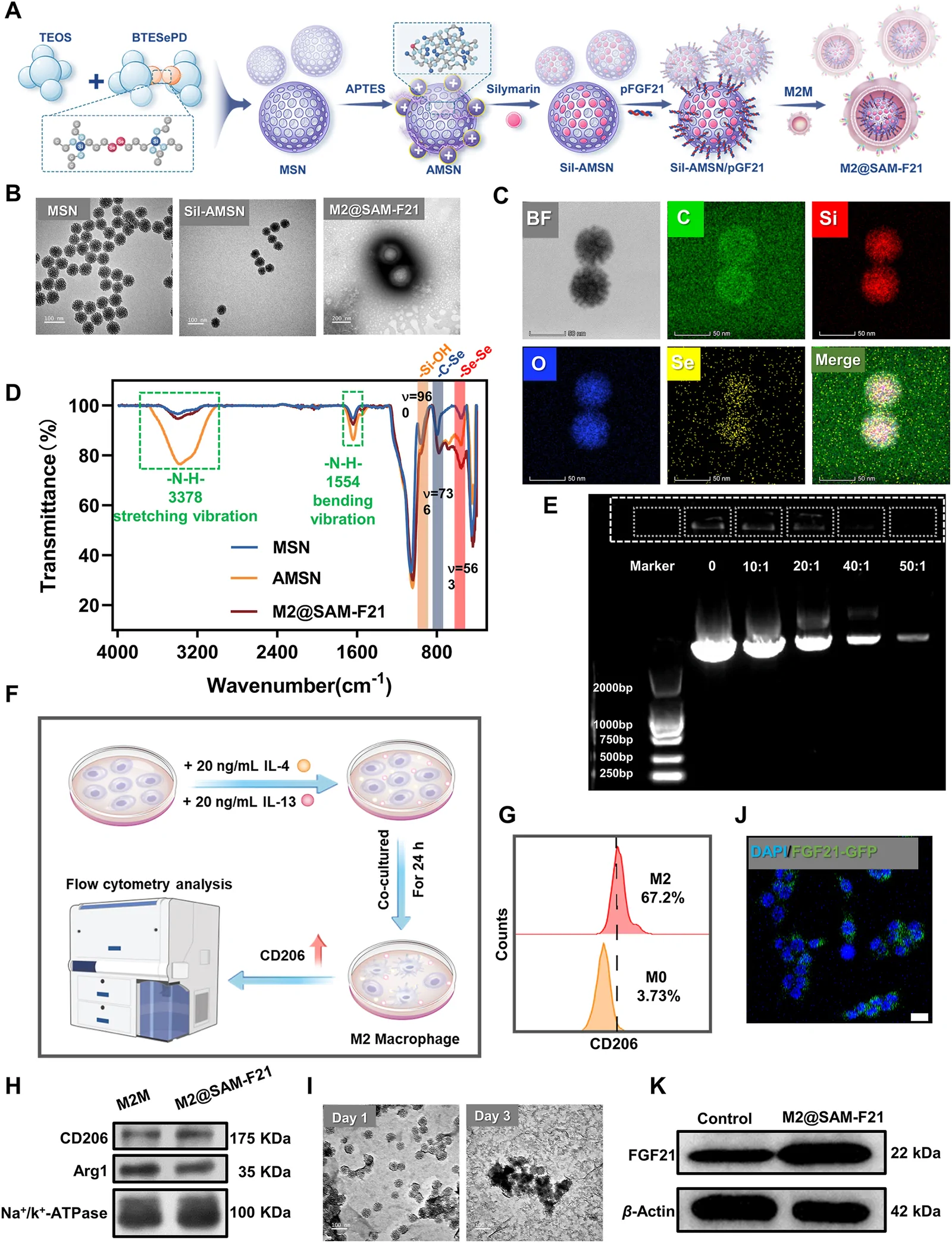
Synthesis and characterization of M2@SAM-F21. (A) Schematic illustration of the preparation process of M2@SAM-F21 nanoparticles. (B) Transmission electron microscopy (TEM) images of MSN, Sil-AMSN, and M2@SAM-F21. (C) Elemental mapping; BF represents bright-field images; C, Si, O, and Se represent different elemental fluorescent signals; and Merge is the overlapping image. Scale bar: 50 nm. (D) Fourier transform infrared (FT-IR) spectra of MSN, AMSN and M2@SAM-F21. (E) AMSN and pFGF21 (10 μg) complexes were formed at mass ratios of 0, 10:1, 20:1, 40:1, and 50:1 (μg/μg) and electrophoresed on 0.7% agarose gels. The loading well from left to right is: molecular weight marker, and samples loaded with 0 μg (control), 100, 200, 400, and 500 μg of AMSN, respectively. (F) Schematic of M2 macrophage polarization. (G) Flow cytometry analysis of macrophage polarization from the M0 to the M2 phenotype. (H) Western blot analysis of M2 macrophage membrane markers in the M2 macrophage membrane group and the M2@SAM-F21 group. Na^+^/K^+^-ATPase was used as a loading control. (I) TEM images of MSN under 100 μmol/L H_2_O_2_ conditions on Days 1 and 3. Scale bar: 100 nm. (J) Immunofluorescence images of cells treated with or without M2@SAM-F21. Blue fluorescence (DAPI) represents cell nuclei, green fluorescence (GFP-tag) represents the expression of FGF21. Scale bar: 20 μm. (K) Western blot analysis of FGF21 and *β*-actin expression levels in the control and M2@SAM-F21-treated groups. *β*-Actin was used as a loading control.

Given that metabolic dysregulation, immune activation, and cellular necrosis during ALI collectively create a microenvironment characterized by elevated ROS^48^ and acidity (pH 5.5–6.8)^49^, material responsiveness was evaluated. As the core component, the MSN exhibited significant structural disintegration upon ROS exposure in TEM imaging (Fig. 1I), indicating that the M2@SAM-F21 formulation retains this sensitivity. *In vitro* release (Supporting Information Fig. S11) studies further demonstrated dual microenvironmental responsiveness: Under physiological conditions (pH 7.4), only about 15% of silymarin was released after 48 h, while the release increased to 85% at pathological pH 5.5. In parallel, in the presence of 100 μmol/L H_2_O_2_, the release reached 92%, which was markedly suppressed to 16% when the antioxidant VC was co-incubated, further confirming the ROS-responsive and on-demand drug release capability of M2@SAM-F21. To assess system functionality, AML-12 cells were co-incubated with M2@SAM-F21. Flow cytometric analysis (Supporting Information Fig. S12) was first performed to quantify transfection efficiency, revealing a high percentage of GFP-positive cells. Confocal microscopy revealed prominent green fluorescence (originating from GFP-tagged pFGF21), which was significantly stronger than that observed in the control or the membrane-free SAM-F21 group (Fig. 1J and Supporting Information Fig. S13). Western blot analysis also revealed significantly increased FGF21 protein expression (Fig. 1K). Collectively, these results demonstrate the successful development of a nanotherapeutic platform exhibiting high targeting specificity, high environmental responsiveness, and efficient transfection capabilities. This platform enables the synergistic codelivery of silymarin and pFGF21 *via* stimuli-responsive drug release properties tailored to disease microenvironments.

### 3.2 *In vitro* ROS scavenging and anti-inflammatory activities of M2@SAM-F21

Oxidative stress serves as a major pathogenic driver of ALI^50^. To evaluate the antioxidant properties of M2@SAM-F21, we selected three typical ROS—superoxide anion radical (O_2_·^−^), hydroxyl radical (·OH), and hydrogen peroxide (H_2_O_2_)—to assess their ROS-scavenging capacity. The results (Fig. 2A–C) demonstrated that M2@SAM-F21 exhibited significant concentration-dependent ROS-scavenging effects. In addition to single-radical scavenging assays, we employed classical 2,2′-azino-bis(3-ethylbenzothiazoline-6-sulfonic acid) (ABTS·^+^) and 1,1-diphenyl-2-picrylhydrazyl (DPPH·) radical scavenging assays. Compared with MSN alone (Supporting Information Figs. S14 and S15), M2@SAM-F21 (Fig. 2D and E) displayed markedly enhanced broad-spectrum antioxidant activity at lower doses, highlighting its potential for treating ROS-related damage at both the cellular and animal levels.

**Figure 2:**
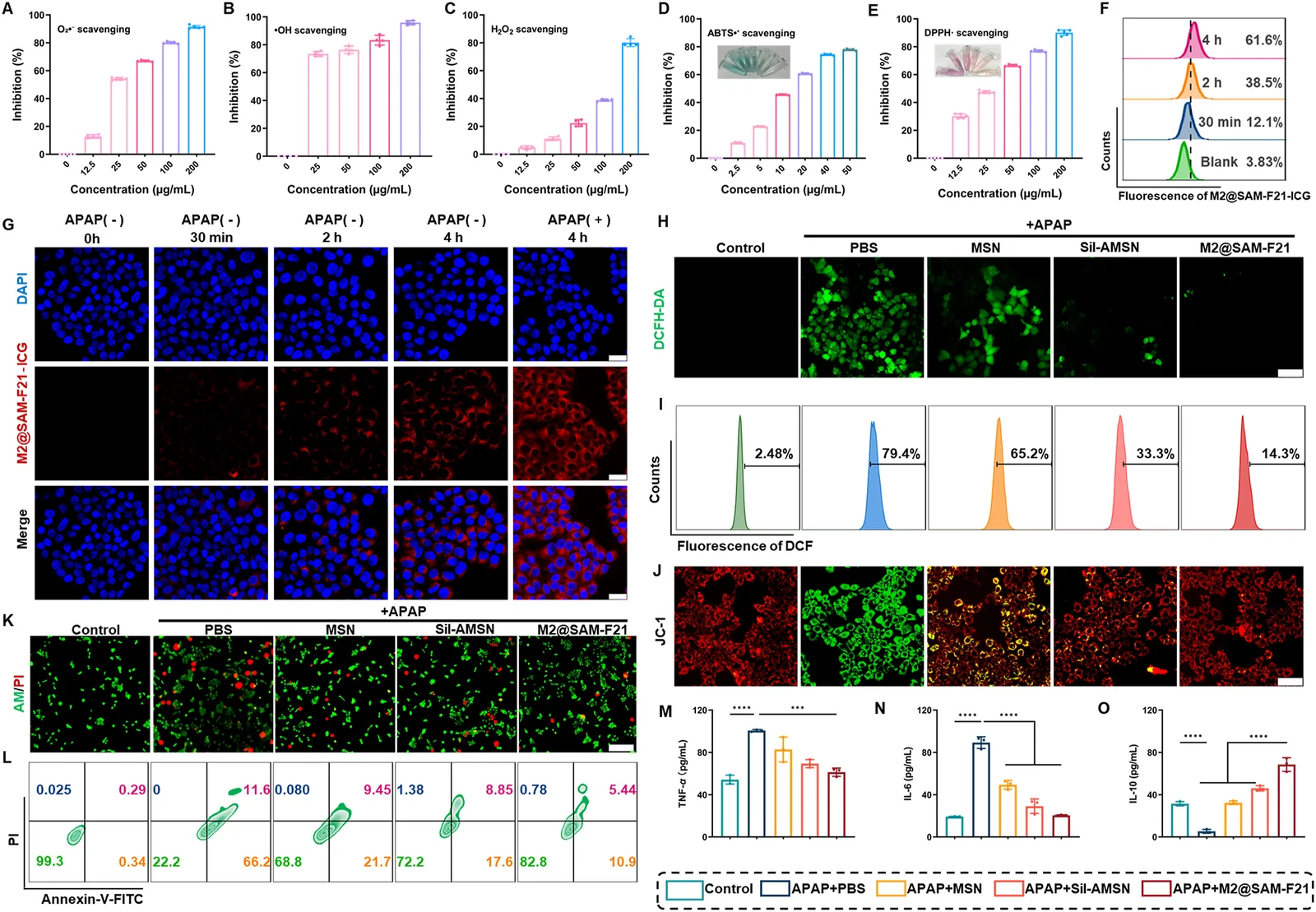
*In vitro* ROS scavenging and anti-inflammatory activities of M2@SAM-F21. (A–E) O_2_‧^−^ (A), ‧OH (B), H_2_O_2_ (C), ABTS‧^+^ (D) and DPPH‧ (E) scavenging abilities of M2@SAM-F21. The data are presented as the mean ± SD from four (B, C) or five (A, D and E) independent replicates. (F) Flow cytometry analysis of the fluorescence intensity of M2@SAM-F21-ICG in AML-12 cells at different time points. (G) Confocal microscopy images of AML-12 cells at different time points after treatment with M2@SAM-F21-ICG. Blue fluorescence (DAPI) indicates cell nuclei, and red fluorescence indicates M2@SAM-F21-ICG. Scale bar: 20 μm. (H) Representative fluorescence images of ROS staining in AML-12 cells under different treatment conditions. Green fluorescence indicates intracellular ROS levels (DCFH-DA staining). Scale bar: 50 μm. (I) Intracellular ROS levels in AML-12 cells were analyzed by flow cytometry. (J) Representative fluorescence images illustrating mitochondrial membrane potential (MMP) in AML-12 cells, assessed using the JC-1 probe. Scale bar: 50 μm. (K) Representative fluorescence images of the viability of AML-12 cells under different treatment conditions. Green fluorescence indicates live cells (calcein-AM), whereas red fluorescence indicates dead cells (PI). Scale bar: 200 μm. (L) Distribution of apoptotic and necrotic cells among AML-12 cells, as shown by FACS analysis. (M–O) Measurement of the concentrations of inflammatory cytokines, including TNF-*α* (M), IL-6 (N), and IL-10 (O), in AML-12 cells. Mean ± SD (*n* = 5), ∗∗∗*P* < 0.001 and ∗∗∗∗*P* < 0.0001, one-way ANOVA.

Prior to biological evaluation, the biocompatibility of the material was assessed using the mouse hepatocyte cell line AML-12, which is known for characteristic liver metabolic functions, and the mouse macrophage line RAW 264.7. Cytotoxicity assays confirmed that MSN, Sil-AMSN, and M2@SAM-F21 maintained excellent cytocompatibility at all the tested concentrations (Supporting Information Figs. S16 and S17). To validate the hepatoprotective effects at the cellular level, a standardized oxidative stress-induced liver injury model was established by exposing AML-12 cells to acetaminophen (APAP). Leveraging the inherent inflammation-homing capacity conferred by abundant cytokine receptors on macrophage membranes, we validated the targeting ability of the biomimetic system using ICG (near-infrared fluorescent dye)-labeled nanoparticles (M2@SAM-F21-ICG). Quantitative flow cytometry analysis revealed that, under physiological conditions, the uptake rate of M2@SAM-F21-ICG by AML-12 cells increased in a dose- and time-dependent manner from 12.1% at 30 min to 61.6% after 4 h of coincubation, confirming basal cellular internalization (Fig. 2F). Strikingly, in the pathological microenvironment of APAP-ALI, the 4-h uptake rate increased to 90.3% (Supporting Information Fig. S18), representing an increase compared with that of the physiological group. Confocal microscopy imaging further corroborated these findings, with APAP-treated cells exhibiting substantially intensified ICG fluorescence signals (Fig. 2G and Supporting Information Fig. S19). To validate the targeting advantage of the M2 macrophage membrane, we introduced SAM-F21 as well as nanoparticles coated with neutrophil membranes (N@SAM-F21) and red blood cell membranes (RB@SAM-F21) as controls. Cellular uptake assays (Supporting Information Fig. S20) demonstrated that, in our established ALI model, the internalization efficiency of M2@SAM-F21 was significantly higher than that of all control groups, thus confirming the superior active targeting capability of the M2 macrophage membrane. As indicated above, the targeting advantage of M2 macrophages is attributed to their intrinsic chemokine receptors. To validate this mechanism, we selected the classic receptor CCR2 for functional investigation. Before coating, M2 membranes were pre-incubated with the CCR2-targeting inhibitor RS102895, and the resulting nanoparticles were then assessed for targeting ability in an inflammatory milieu. The results (Supporting Information Fig. S21) showed that cellular uptake was significantly reduced when the receptor was blocked, confirming that the targeting effect is indeed mediated by specific chemokine receptors present on the M2 macrophage membrane. These findings unequivocally demonstrate that inflammation-dependent targeted augmentation occurred. Collectively, these results establish that M2@SAM-F21 exhibits spatiotemporal specificity for precise accumulation at inflammatory loci. This active targeting capability is paramount for enhancing the therapeutic efficacy and safety profile of nanomedicines.

Intracellular ROS levels were first detected using the fluorescent probe 2′,7′-dichlorodihydrofluorescein diacetate (DCFH-DA). Both fluorescence microscopy and flow cytometry (Fig. 2H and I) confirmed the hierarchical ROS-scavenging capacity across the treatment groups. AML-12 cells in the model group exhibited excessive ROS accumulation, while pretreatment with equivalent doses of MSN, Sil-AMSN, or M2@SAM-F21 progressively attenuated intracellular oxidative stress. Crucially, the biomimetic M2@SAM-F21 system restored ROS levels to near-physiological baseline levels (Supporting Information Fig. S22). The superior efficacy was visually confirmed through diminished DCF fluorescence intensity, as observed on confocal images, which directly confirmed the cooperative action of each component of the system in abating oxidative stress. Mitochondria, as both major sources and targets of ROS, play a central role in the progression of ALI. The 5,5′,6,6′-tetrachloro-1,1′,3,3′-tetraethyl-imidazolylcarbocyanine (JC-1) probe assay revealed that APAP treatment significantly reduced the mitochondrial membrane potential (Fig. 2J and Supporting Information Fig. S23). While MSN and Sil-AMSN only partially restored the JC-1 aggregate/monomer ratio, M2@SAM-F21 more effectively stabilized the membrane potential (Supporting Information Fig. S24). Since cell death is the ultimate outcome of APAP-induced liver injury, we evaluated cytoprotective effects using calcein-AM/PI double staining, which revealed that M2@SAM-F21 treatment significantly reduced cell death (Fig. 2K and Supporting Information Fig. S25). This protective effect was further corroborated by Annexin V/PI apoptosis assays, which demonstrated that M2@SAM-F21 markedly suppressed APAP-induced apoptosis (Fig. 2L), with quantitative analysis indicating a significant decrease in both early and late apoptotic cell populations (Supporting Information Fig. S26).

Inflammation plays a central role in APAP-ALI, with tumor necrosis factor-alpha (TNF-*α*) and interleukin-6 (IL-6) serving as major proinflammatory cytokines that are often excessively released during hepatic damage, thereby amplifying inflammatory cascades and aggravating hepatocyte injury. In contrast, interleukin-10 (IL-10) functions as a key anti-inflammatory cytokine that counteracts these effects. Subsequent measurements of typical cytokine expression levels revealed that M2@SAM-F21 significantly inhibited the APAP-induced overproduction of TNF-*α* and IL-6 while increasing IL-10 levels (Fig. 2M–O). These results conclusively demonstrate that M2@SAM-F21 effectively mitigates APAP-induced hepatocyte damage through synergistic antioxidant, mitochondrial protective, and anti-inflammatory mechanisms.

### 3.3 Immune balance capacity of M2@SAM-F21

Macrophages are strategically distributed throughout the body and play a crucial role in maintaining immune homeostasis by clearing invading pathogens and harmful substances. The M1 phenotype is associated with strong proinflammatory activity, characterized by the production of proinflammatory cytokines and the recruitment of immune cells to eliminate pathogens. However, an excessive M1 response, particularly when accompanied by a reduction in anti-inflammatory M2 macrophages and the infiltration of other immune cells, can result in a dysregulated inflammatory environment^51^. This imbalance contributes to sustained immune activation and aggravates hepatic tissue damage, thereby accelerating the progression of ALI.

Given that lipopolysaccharide (LPS) specifically activates M1 macrophages, thereby mimicking the inflammatory response and immune imbalance observed in ALI, we utilized an LPS-induced RAW264.7 macrophage model to investigate inflammatory responses and immune cell activation. As shown in Fig. 3A and Supporting Information Fig. S27, LPS stimulation significantly upregulated the expression of CD86, a key marker associated with M1 macrophage polarization, in RAW264.7 cells. Notably, treatment with M2@SAM-F21 not only significantly reduced CD86 expression but also increased the expression of CD206 (Fig. 3B and Supporting Information Fig. S28), a key marker associated with M2 macrophage polarization. Flow cytometric profiles aligned with these observations (Fig. 3C and D, Supporting Information Figs. S29 and S30). These results were confirmed at the protein level by Western blot analysis (Fig. 3E). Statistical evaluation revealed that, compared with that in the control group, CD86 expression in the LPS-treated group increased 1.4-fold, whereas M2@SAM-F21 intervention reversed CD86 expression 0.9-fold. Conversely, CD206 expression was significantly increased relative to that in the model group (Fig. 3F). When compared with N@SAM-F21 or RB@SAM-F21, M2@SAM-F21 still demonstrated the most pronounced effect in promoting M2 macrophage polarization (Supporting Information Fig. S31). Collectively, these results demonstrate that M2@SAM-F21 mitigates the progression of ALI by modulating macrophage polarization and reversing the immune imbalance.

**Figure 3:**
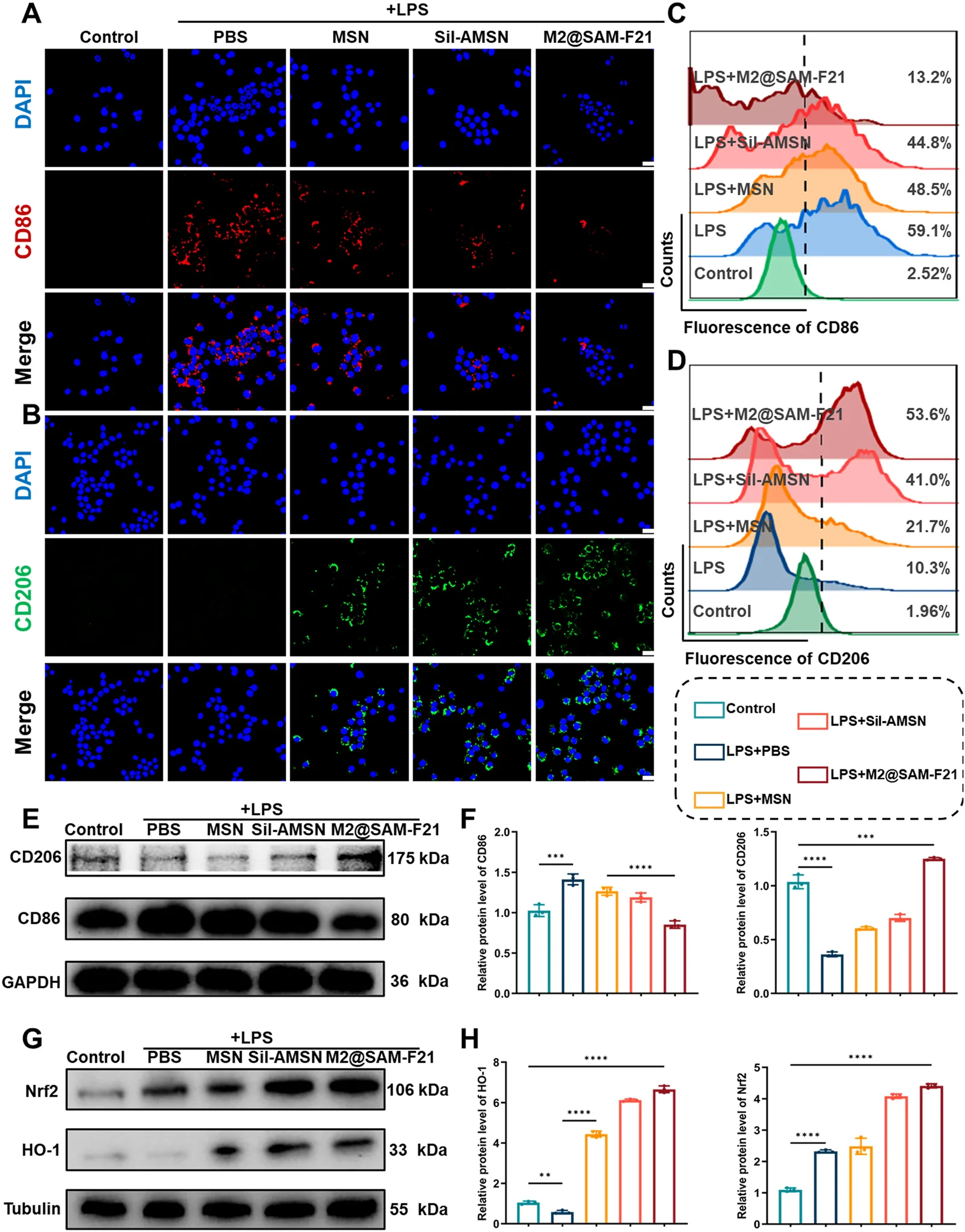
Immune balance capacity of M2@SAM-F21. (A) Immunofluorescence analysis of LPS-stimulated RAW264.7 macrophages; nuclei are stained with DAPI (blue), and the M1 polarization marker CD86 is red. Scale bar: 20 μm. (B) Immunofluorescence analysis of LPS-stimulated RAW264.7 macrophages; nuclei are stained with DAPI (blue), and the M2 polarization marker CD206 is green. Scale bar: 20 μm. (C) Intracellular CD86 levels in RAW 264.7 cells were analyzed by flow cytometry. (D) Intracellular CD206 levels in RAW 264.7 cells were analyzed by flow cytometry. (E) Western blot analysis of CD206 and CD86 levels in different groups. (F) Quantitative analysis of the relative protein levels of CD86 (left) and CD206 (right). (G) Western blot analysis of Nrf2 and HO-1 expression levels in different groups. (H) Quantitative analysis of the relative protein levels of Nrf2 (left) and HO-1 (right). Mean ± SD (*n* = 3), ∗∗*P* < 0.01, ∗∗∗*P* < 0.001, ∗∗∗∗*P* < 0.0001 and ns indicates no significance, One-way ANOVA.

Building on these findings, further mechanistic exploration focused on identifying the molecular pathways responsible for the observed immunomodulatory and antioxidant effects. The Nrf2/HO-1 pathway has been shown to confer multiorgan protection and plays a pivotal regulatory role in various ALI models^52^^,^^53^. Nrf2, a master regulator of the cellular redox balance, is highly expressed in metabolically active organs such as the liver. Upon activation, Nrf2 is translocated into the nucleus and binds to the antioxidant response element (ARE) in the promoter regions of target genes. This binding initiates the transcription of downstream phase II detoxifying/antioxidant enzymes, including HO-1, the catalytic subunit of glutamate-cysteine ligase (GCLC), and NAD(P)H quinone oxidoreductase 1 (NQO1)^54^^,^^55^. Given that Nrf2-mediated elimination of ROS constitutes the molecular basis of its anti-inflammatory effects, the activation of the Nrf2 pathway and its associated antioxidant enzymes is considered crucial for treating liver injury. To elucidate the mechanism by which M2@SAM-F21 alleviates oxidative stress and inflammation, we assessed the expression levels of Nrf2 and its key downstream effector, HO-1. Western blot analysis (Fig. 3G) revealed that Nrf2 protein levels were significantly higher in M2@SAM-F21-treated RAW264.7 cells than in both the LPS model cells and the untreated control cells. Furthermore, concomitant significant upregulation of HO-1 expression (*P* < 0.0001) was observed, indicating the effective activation of Nrf2 transcriptional activity (Fig. 3H). These findings confirm that M2@SAM-F21 exerts hepatoprotective effects by enhancing the activity of the Nrf2/HO-1 pathway, thereby suppressing proinflammatory responses and ameliorating oxidative stress.

### 3.4 Metabolic regulatory capacity of M2@SAM-F21

Silymarin has long been recognized for its hepatoprotective properties, including its antioxidant and lipid-lowering effects^56^. In parallel, FGF21 is primarily synthesized and secreted into the bloodstream by the liver, with additional expression occurring in extrahepatic tissues such as white adipose tissue, brown adipose tissue, and skeletal muscle. As a key metabolic regulator, FGF21 plays a central role in maintaining glucose homeostasis, enhancing insulin sensitivity, and promoting fatty acid oxidation, establishing its importance as a crucial molecule in the regulation of glucose and lipid metabolism^57^. On the basis of the complementary roles of silymarin and FGF21, we hypothesized that the engineered carrier M2@SAM-F21 not only possesses previously documented anti-inflammatory, antioxidant, and immunomodulatory capabilities but also may confer lipid metabolism regulatory functions.

To evaluate the physiological effects of M2@SAM-F21 in the context of ALI, an *in vitro* model of alcohol-induced hepatotoxicity was established by treating AML-12 cells with ethanol, which effectively induced characteristic lipid accumulation associated with alcoholic liver injury. To evaluate the interventional effect of M2@SAM-F21 on ethanol-induced lipid accumulation, multimodal lipid staining techniques were employed. Staining with Oil Red O, a next-generation Sudan lipid dye with established diagnostic utility for visualizing atherosclerotic plaques and hepatic/muscular lipid droplets, revealed significantly higher lipid droplet counts in ethanol-treated AML-12 cells than in control cells (Fig. 4A), whereas M2@SAM-F21 treatment markedly reduced lipid accumulation. Notably, the pure antioxidant carrier MSN, which possesses antioxidant activity but lacks specific lipid-lowering functionality, failed to significantly decrease lipid droplet density, whereas M2@SAM-F21 demonstrated optimal lipid-clearing efficacy. This phenomenon was dually validated through Nile red (fluorescently bound to lipid moieties) (Fig. 4B) and BODIPY 493/503 (lipophilic fluorescent probe) staining (Fig. 4C): for both probes, there was substantially diminished fluorescence intensity in the M2@SAM-F21 group compared with the model group (Fig. 4D–F). Collectively, these findings confirm that M2@SAM-F21 not only possesses previously verified anti-inflammatory, antioxidant, and immunomodulatory functions but also achieves lipid metabolic reprogramming *via* its codelivery system. The core mechanism involves the successful expression of pFGF21 in hepatocytes and its synergistic action with silymarin, which significantly regulates lipid metabolism and alleviates lipid accumulation in an *in vitro* alcoholic liver injury model, thereby reversing lipotoxic injury through multitargeted pathways.

**Figure 4:**
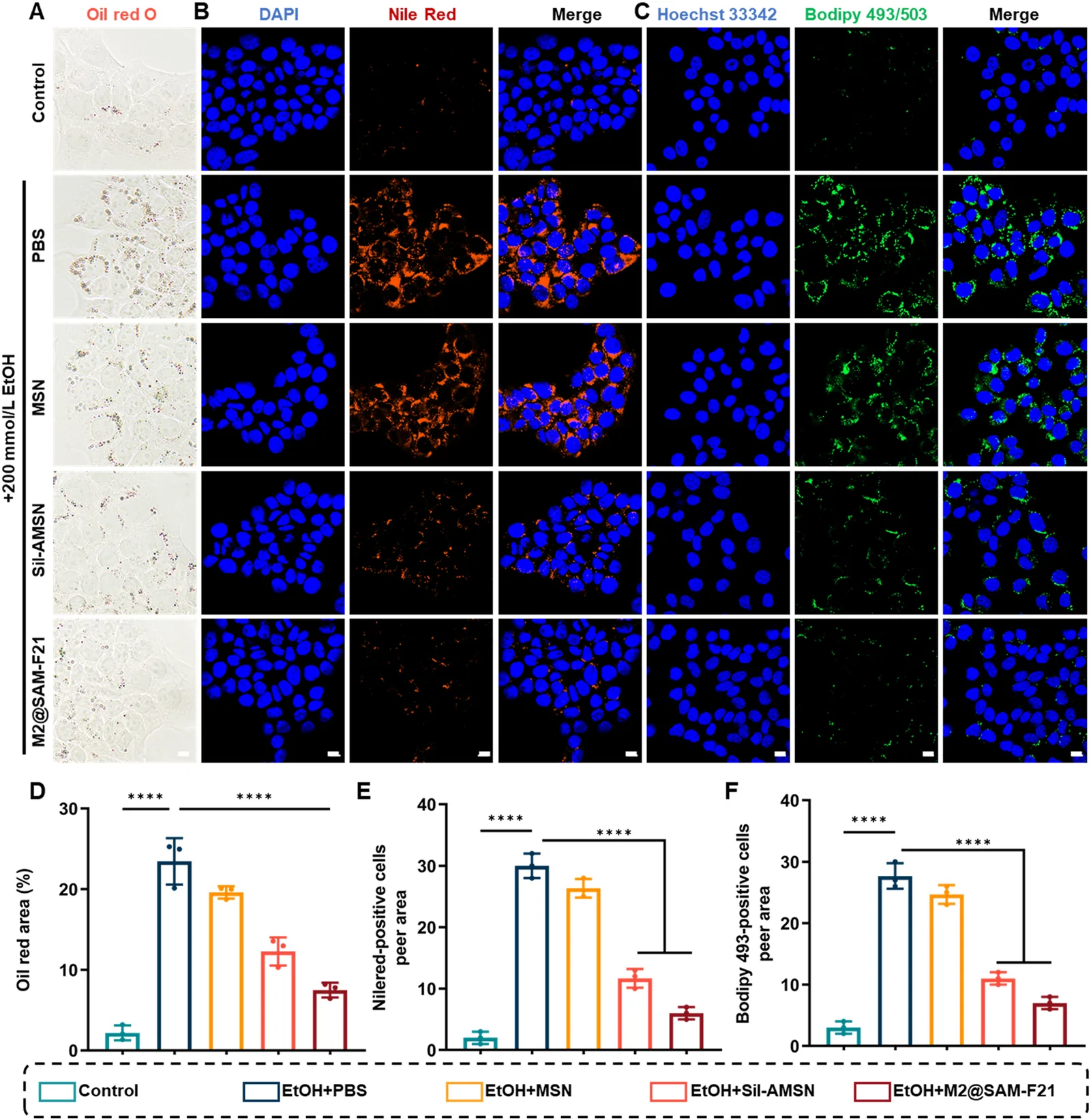
Metabolic regulatory capacity of M2@SAM-F21. (A) Images of Oil Red O-stained cells treated with different agents and 200 mmol/L EtOH. The images show lipid accumulation in the cells. Scale bar: 100 μm. (B) Immunofluorescence images of 200 mmol/L EtOH-stimulated cells stained with DAPI (blue, cell nuclei) and Nile Red (red, lipid droplets). Scale bar: 10 μm. (C) Immunofluorescence images of 200 mmol/L EtOH-stimulated cells stained with Hoechst 33342 (blue, cell nuclei) and BODIPY 493/503 (green, lipid droplets). Scale bar: 10 μm. (D–F) Statistical graphs showing the relative area of the Oil Red O-stained area (D), Nile red-positive cells (E) and BODIPY 493-positive cells (F) in each group. Mean ± SD (*n* = 3), and ∗∗∗∗*P* < 0.0001, one-way ANOVA.

### 3.5 *In vivo* biosafety and targetability assessment of M2@SAM-F21

Building upon the validated hepatoprotective efficacy and inflammation-targeting advantages of M2@SAM-F21 *in vitro*, this study systematically assessed the *in vivo* biocompatibility and targeted delivery performance of M2@SAM-F21. Following a single high-dose intravenous administration, histopathological analyses of major organs (heart, liver, spleen, lungs, and kidneys) on Days 1 and 14 post-injection revealed no pathological lesions (Fig. 5A and Supporting Information Fig. S32). Serum biochemical assays (Fig. 5B and C) revealed that the levels of liver function markers aspartate—aminotransferase (AST) and alanine aminotransferase (ALT), as well as renal function parameters—creatinine (CRE) and blood urea nitrogen (BUN), were not significantly different from those in saline-injected controls (*P* > 0.05). Complete blood analysis (Supporting Information Figs. S33 and S34) confirmed that the hematological parameters remained within normal reference ranges (*P* > 0.05). Hemolysis assays (Supporting Information Fig. S35) indicated minimal erythrocyte damage, with a hemolysis ratio of only 3.2 ± 0.3% at a concentration of 200 μg/mL, which is below the 5% safety threshold specified in ISO 10993-5. Taken together, these findings demonstrate the favorable *in vivo* biosafety profile of M2@SAM-F21, including its nonhemolytic nature at therapeutic concentrations. This established safety foundation supports intravenous administration and facilitates subsequent therapeutic efficacy evaluations in disease models.

**Figure 5:**
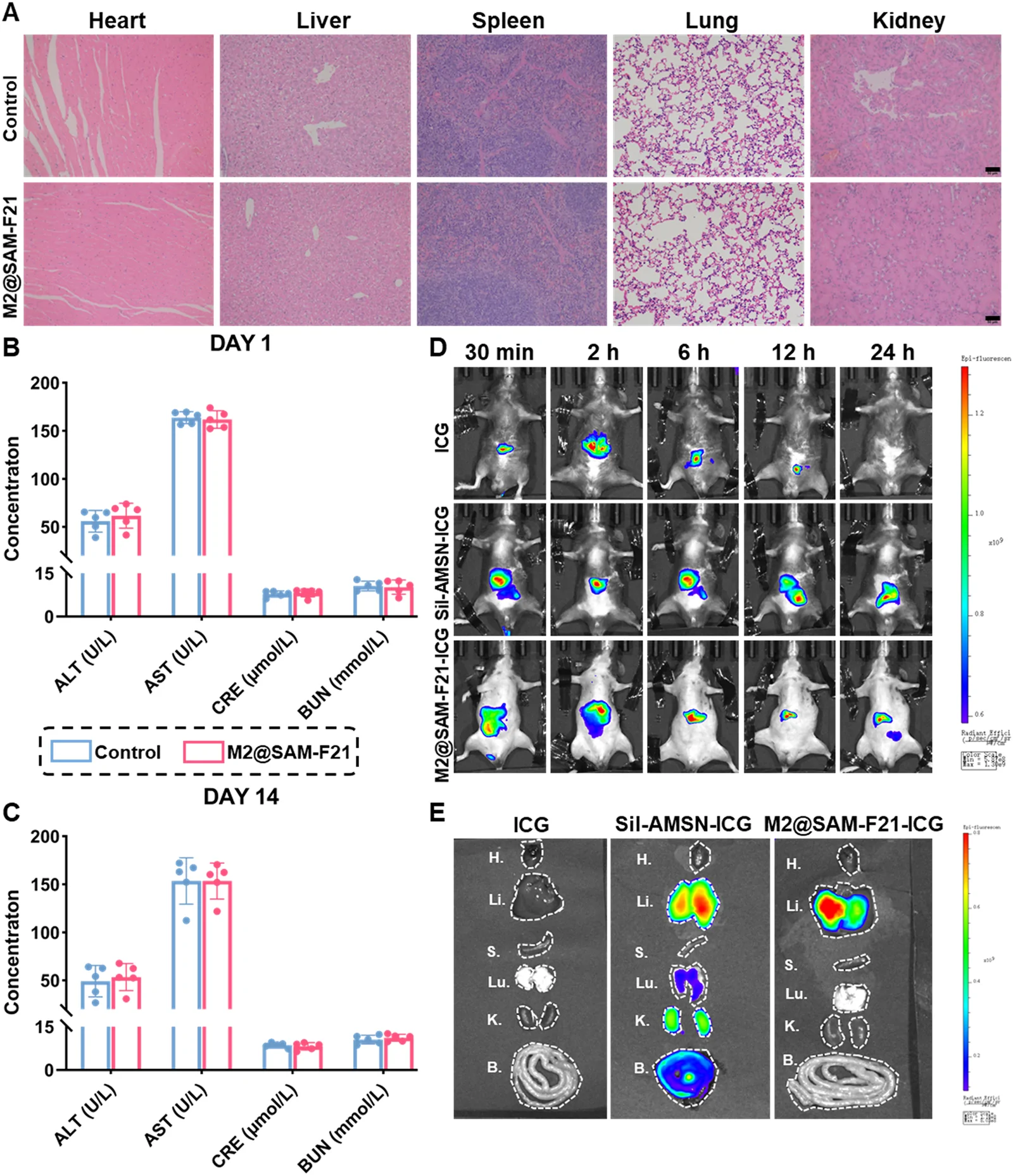
*In vivo* biosafety and targetability assessment of M2@SAM-F21. (A) Evaluation of the *in vivo* toxicity of M2@SAM-F21 to major organs (heart, liver, spleen, lung and kidney) at 14 days after intravenous administration. Scale bar: 50 μm. (B, C) Serum levels of liver function indicators (ALT and AST) and renal function parameters (CRE and BUN) in normal mice (control group) and in mice intravenously injected with M2@SAM-F21 1 day (B) or 14 days (C) after injection. (Mean ± SD, *n* = 5). (D) *In vivo* fluorescence imaging of mice at different time points (30 min, 2, 6, 12, and 24 h) after the tail-vein injection of ICG, Sil-AMSN-ICG, or M2@SAM-F21-ICG. The fluorescence intensity in the liver region is shown. (E) Representative images of *ex vivo* fluorescence imaging of major organs (H: heart, Li: liver, S: spleen, Lu: lung, K: kidney and B: bowel).

Given the pivotal role of targeted delivery in nanomedicine efficacy, near-infrared imaging of ICG-labeled nanoparticles was performed using APAP-ALI models. Whole-animal imaging revealed initial hepatic accumulation in all groups at 2 h post-injection. Temporal tracking revealed the rapid clearance of free ICG, whereas nanoparticle formulations showed prolonged retention. A critical observation emerged at 12 h: noncoated nanoparticles (Sil-AMSN-ICG) presented markedly diminished liver signals with concurrent extrahepatic migration, whereas M2@SAM-F21-ICG maintained progressive hepatic accumulation through 24 h (Fig. 5D). *Ex vivo* organ imaging (Fig. 5E and Supporting Information Fig. S36) at 24 h confirmed sustained liver-specific signal intensity of M2@SAM-F21-ICG, which significantly outperformed the control formulations with regard to targeted accumulation. Collectively, these results demonstrate that M2@SAM-F21-ICG exhibits significantly enhanced and specific hepatic targeting and accumulation compared with both free ICG and uncoated Sil-AMSN-ICG nanoparticles. This *in vivo* targeting profile aligns with the *in vitro* targeting data, providing strong corroborative evidence that the M2 macrophage membrane coating confers M2@SAM-F21 with superior inflammatory tropism.

### 3.6 *In vivo* therapeutic effect of M2@SAM-F21 against ALI

Having established the *in vitro* hepatoprotective, inflammation-targeting, and multiregulatory functions of M2@SAM-F21, we next assessed its therapeutic efficacy in a mouse model of alcoholic liver injury (EtOH-ALI). Following EtOH-ALI induction using the Lieber-DeCarli ethanol liquid diet^58^^,^^59^, ALI mice were randomized into four groups: model control group, MSN-treated group, Sil-AMSN-treated group, and M2@SAM-F21-treated group. Eight hours after a high-dose ethanol gavage, the mice were switched to standard chow and administered the corresponding formulations *via* daily intravenous injections for 7 consecutive days. The samples were collected 9 h after the final treatment (Fig. 6A). Serological analysis (Fig. 6B–E) revealed significantly elevated ALT/AST levels accompanied by abnormal TC/TG accumulation in the model group, confirming ethanol-induced hepatic damage with lipid metabolic dysregulation. In contrast, the M2@SAM-F21 treatment group exhibited comprehensive improvement, with ALT, AST, TG, and TC levels decreasing to near-normal ranges, and the efficacy of the M2@SAM-F21 system was markedly superior to that of single-component systems. Histopathological assessment (H&E staining) validated those findings: the model group displayed characteristic alcoholic liver injury features, including cytoplasmic vacuolization, nuclear pyknosis, and microvesicular steatosis, whereas the M2@SAM-F21 group maintained lobular architecture integrity with substantially reduced steatotic areas (Fig. 6F and Supporting Information Fig. S37). This evidentiary cascade demonstrates that M2@SAM-F21 effectively reversed ethanol-induced hepatic pathological progression through a synergistic codelivery mechanism.

**Figure 6:**
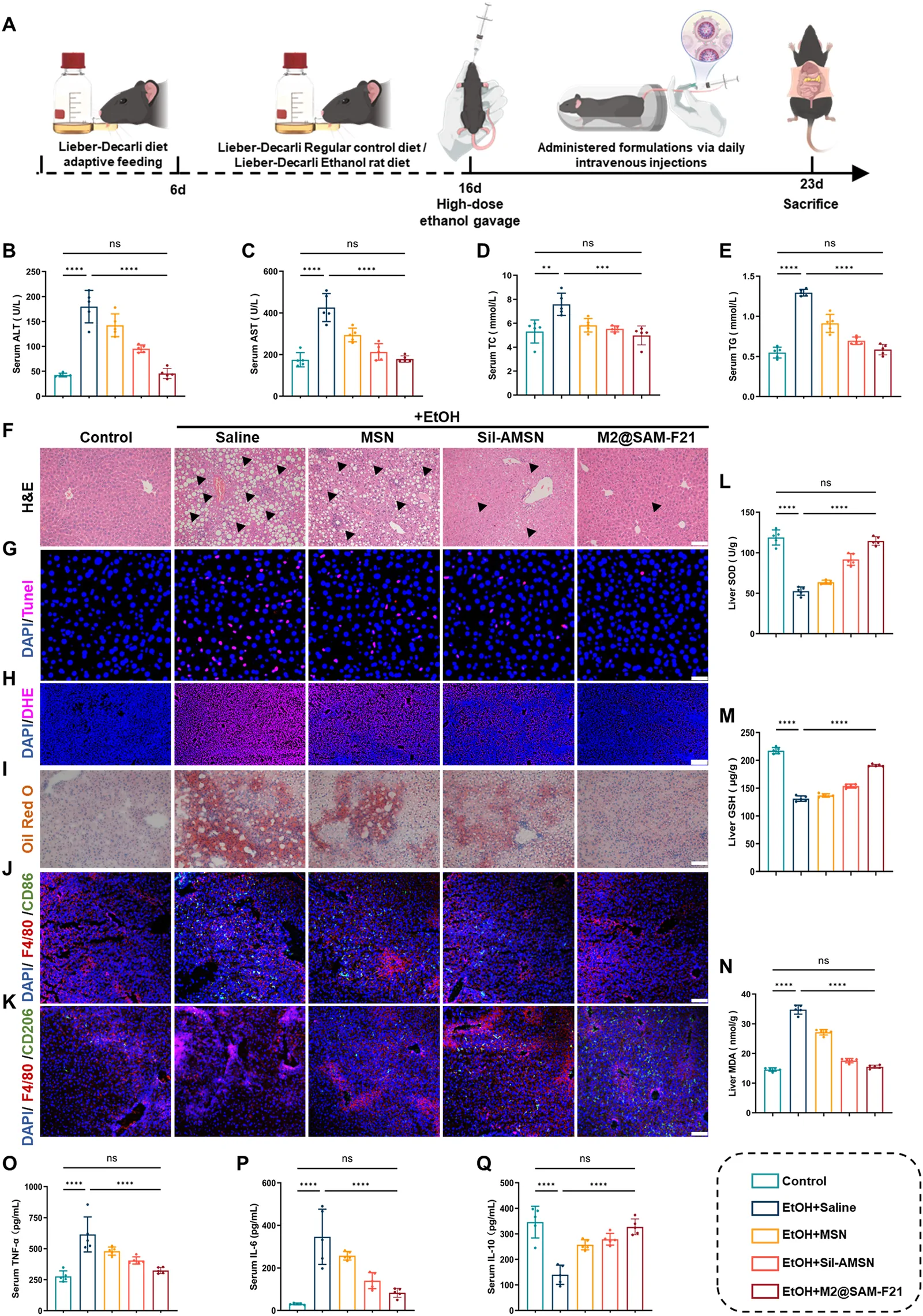
*In vivo* therapeutic effect of M2@SAM-F21 against ALI. (A) Schematic diagram of the experimental timeline for the Lieber–DeCarli diet-induced alcoholic liver injury mouse model. The timeline is divided into four phases: adaptive feeding, regular control diet/ethanol rat diet, normal diet and cure, and sacrifice. (B–E) Serum levels of ALT (B), AST (C), TC (D), and TG (E) in different groups during the experimental period. (F–K) Representative H&E staining (F), TUNEL assay (G), DHE staining (H), Oil Red O staining (I) and immunofluorescence staining (F4/80/CD86 (J) and F4/80/CD206 (K)) images of liver tissues from different groups. Nuclei were stained with DAPI (blue), macrophages were labeled with F4/80 (red), and M1 or M2 phenotypes were marked with CD86 (green, panel J) or CD206 (green, panel K), respectively. Scale bar: 50 μm. Arrows indicate specific pathological features. (L–N) Liver GSH (L), MDA (M) and SOD (N) levels in different groups. (O–Q) Serum levels of TNF-*α* (O), IL-6 (P), and IL-10 (Q) in different groups. (Mean ± SD, *n* = 5. ∗*P* < 0.05, ∗∗*P* < 0.01, ∗∗∗*P* < 0.001, ∗∗∗∗*P* < 0.0001 and ns means not significant; one-way ANOVA).

To confirm the functional delivery of the system *in vivo*, we first analyzed FGF21 expression during the treatment by Western blot. The results showed that although FGF21 protein levels were compensatorily elevated in the EtOH-ALI group, they were further significantly increased after M2@SAM-F21 treatment (Supporting Information Fig. S38). This observation was also supported by immunohistochemical (IHC) analysis of liver tissues (Supporting Information Fig. S39). Together, these data indicate that M2@SAM-F21 not only exhibits good transfection efficiency at the cellular level but also effectively delivers and promotes high-level expression of FGF21 protein *in vivo*, thereby providing a mechanistic basis for the subsequent therapeutic effects observed. Alcohol metabolism in the body generates substantial amounts of free radicals and other intermediates that disrupt systemic homeostasis and interfere with normal cellular metabolism, thereby inducing hepatocyte disease. Liver sections were subjected to immunofluorescence staining to assess the antioxidant efficacy of M2@SAM-F21. Terminal deoxynucleotidyl transferase dUTP nick end labeling (TUNEL) revealed that M2@SAM-F21 treatment significantly reduced the number of apoptotic hepatocytes (Fig. 6G and Supporting Information Fig. S40). Concurrently, dihydroethidium (DHE) staining to assess hepatic superoxide levels confirmed the critical role of ROS in the development of EtOH-ALI and demonstrated the *in vivo* antioxidant effect of M2@SAM-F21, as M2@SAM-F21 treatment significantly reversed the elevated DHE fluorescence intensity, indicating the effective mitigation of oxidative damage (Fig. 6H and Supporting Information Fig. S41). To systematically evaluate hepatocellular lipid accumulation, Oil Red O staining of liver sections was performed. Subsequent image analysis demonstrated that M2@SAM-F21 significantly alleviated alcohol-induced lipid deposition (Fig. 6I). Further characterization of the hepatic immune microenvironment was performed *via* macrophage immunofluorescence staining and flow cytometry (Fig. 6J and K, Supporting Information Figs. S42–S44). Immunofluorescence analysis revealed a substantial expansion of F4/80^+^/CD86^+^ M1 macrophages in the model group, a finding quantitatively confirmed by flow cytometry, collectively confirming alcohol-induced immune homeostasis disruption. In contrast, the M2@SAM-F21 treatment group presented significantly elevated proportions of F4/80^+^/CD206^+^ M2 macrophages concomitant with a reduced M1 population, as consistently demonstrated by both imaging and quantitative flow cytometric data. Mechanistically, this shift in macrophage polarization is driven by the activation of the Nrf-2/HO-1 antioxidative pathway, a mechanism we previously established *in vitro*. The present *in vivo* IHC staining (Supporting Information Fig. S45) further confirmed that M2@SAM-F21 treatment significantly up-regulated the expression of both Nrf-2 and HO-1 in liver tissues, thereby extending the cellular mechanism to the whole-animal level and providing a molecular basis for the observed immunomodulation. Those collectively validating the therapeutic reversal of the immune imbalance.

Furthermore, such liver diseases are characterized by increased hepatic malondialdehyde (MDA) content and decreased activity of enzymatic antioxidants such as glutathione (GSH) and superoxide dismutase (SOD)^60^, ^61^, ^62^. Enzyme activity assays, as shown in Fig. 6L–N, confirmed that, compared with those from the control group, liver tissue from the ethanol model group was in an oxidative state, exhibiting significantly reduced GSH and SOD activity alongside significantly elevated MDA levels. M2@SAM-F21 treatment reversed those alterations, significantly increasing SOD and GSH activity and significantly reducing hepatic MDA levels compared with those observed in the model group. In summary, these results underscore the efficacy of M2@SAM-F21 treatment in ameliorating alcohol-induced hepatic oxidative stress injury. In the early stages of alcoholic liver injury, TNF-*α* and IL-6 act as key proinflammatory cytokines triggering the inflammatory cascade. As shown in Fig. 6O‒Q, the serum levels of TNF-*α* and IL-6 were significantly higher in the alcohol model group than in the control group, whereas the level of the anti-inflammatory cytokine IL-10 was lower. Following M2@SAM-F21 treatment, the TNF-*α* and IL-6 levels significantly decreased, whereas the IL-10 level markedly increased. These results demonstrate that M2@SAM-F21 effectively prevents and alleviates alcohol-induced hepatic inflammatory damage by modulating inflammatory cytokine release, suppressing proinflammatory responses and enhancing anti-inflammatory responses.

Consistent with previous findings, M2@SAM-F21 exhibited significant therapeutic efficacy in the APAP-induced ALI (APAP-ALI) model mice (Supporting Information Fig. S46). M2@SAM-F21 administration reversed APAP-induced hepatomegaly, as evidenced by a normalized liver-to-body weight ratio (Supporting Information Fig. S47). This macroscopic recovery was corroborated by histological and biochemical assessments, which revealed a substantial reduction in parenchymal damage and the restoration of hepatic function (Supporting Information Figs. S48–S50). Together, these findings establish that M2@SAM-F21 synergistically ameliorates steatosis, oxidative stress, inflammatory cascades, and immune dysregulation, demonstrating translational potential as a multitarget therapeutic strategy against ALI.

### 3.7 Proteomics-based investigation of the therapeutic mechanism of M2@SAM-F21

Proteomic data provide a critical foundation for elucidating therapeutic mechanisms and guiding clinical research. To investigate the underlying mechanisms of M2@SAM-F21 in treating ALI, we performed proteomic analyses using liver samples from three mouse groups: the control group, ALI model group, and M2@SAM-F21-treated group (abbreviated as M2@SAM-F21). A total of 761 and 1009 proteins (Fig. 7A and B) were identified as differentially expressed in the control *vs.* ALI comparison and ALI *vs.* M2@SAM-F21 comparison, respectively.

**Figure 7:**
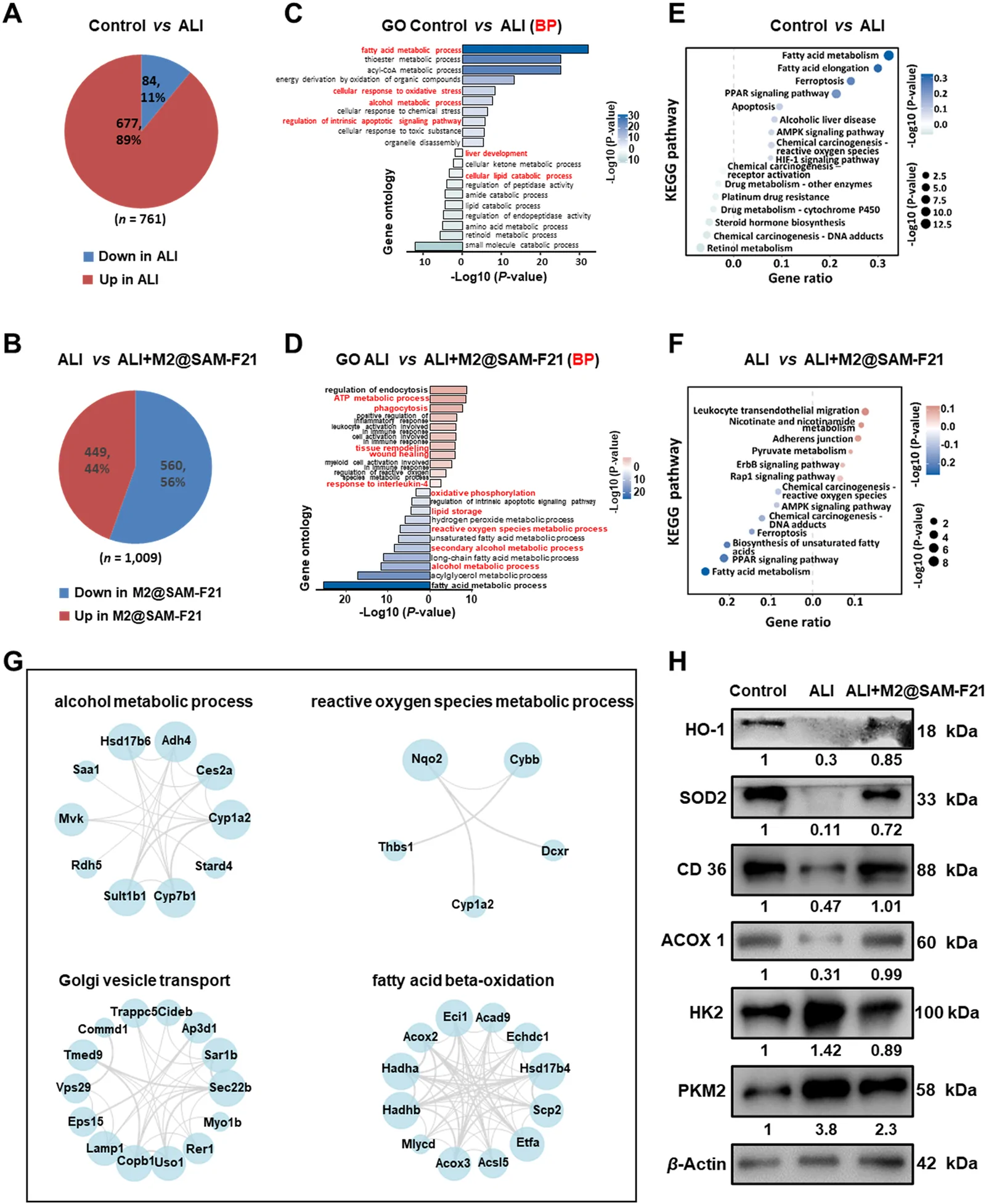
Proteomics-based investigation of the therapeutic mechanism of M2@SAM-F21. (A) Pie chart showing the distribution of differentially expressed proteins in ALI. (B) Pie chart showing the distribution of differentially expressed proteins after M2@SAM-F21 treatment. (C) GO-BP analysis of the upregulated or downregulated proteins in the Control group-ALI group comparison. (D) GO-BP analysis of the upregulated or downregulated proteins in the ALI group-M2@SAM-F21 group comparison. (E) KEGG pathway analysis of the differentially expressed proteins in the Control group-ALI group comparison. (F) KEGG pathway analysis of the differentially expressed proteins in the ALI group-M2@SAM-F21 group comparison. (G) Protein–protein interaction network analysis of differentially expressed genes in the Control, ALI and M2@SAM-F21 treatment groups. Nodes represent proteins, and edges represent interactions. The network focuses on processes such as alcohol metabolism, reactive oxygen species metabolism, and fatty acid beta-oxidation. (H) Western blot analysis of HO-1, SOD2, CD36, ACOX1, HK2, PKM2, and *β*-actin expression levels in different groups. *β*-Actin was used as a loading control. The molecular weights of the proteins are indicated on the right.

Gene Ontology (GO) analysis of the biological processes (BP), cellular components (CC), and molecular functions (MF) of the identified differentially expressed proteins was performed. Fig. 7A shows that among the proteins differentially expressed between the ALI and control groups, 677 were upregulated, whereas 87 were downregulated. The BP terms significantly enriched in the ALI group included fatty acid metabolic process, energy derivation by the oxidation of organic compounds, cellular response to oxidative stress, alcohol metabolic process, cellular response to chemical stress, and regulation of the intrinsic apoptotic signaling pathway (Fig. 7C). These enriched processes are consistent with the pathological characteristics of ALI, including lipid accumulation, oxidative stress, and hepatocyte damage. At the MF level (Supporting Information Fig. S51), significantly upregulated pathways involved oxidoreductase activity acting on the CH–OH group of donors (with NAD or NADP as acceptors), fatty acyl-CoA hydrolase activity, ATP hydrolysis activity, and iron ion binding, which are closely associated with oxidative stress and lipid metabolism dysregulation. CC analysis (Supporting Information Fig. S52) indicated that most of the differentially expressed proteins were localized in the cytosol, suggesting potential involvement in mitochondrial dysfunction. Compared with those in the ALI group, 449 proteins were upregulated and 560 were downregulated in the M2@SAM-F21 intervention group (Fig. 7B). GO enrichment analysis revealed that M2@SAM-F21 treatment significantly upregulated the expression of genes involved in BPs such as ATP metabolic processes, positive regulation of the inflammatory response, cell activation involved in the immune response, and the response to interleukin-4 signaling; concurrently, it significantly downregulated the expression of genes involved in processes such as fatty acid metabolic processes, alcohol metabolic processes, reactive oxygen species metabolic processes, lipid storage, regulation of the intrinsic apoptotic signaling pathway, and oxidative phosphorylation (Fig. 7D). These results demonstrate that M2@SAM-F21 primarily alleviates ALI pathological damage by suppressing oxidative stress, inflammatory responses, and abnormal lipid metabolism. Similarly, further MF and CC analyses (Supporting Information Figs. S53 and S54) revealed that the treatment group was significantly enriched in functions such as metal ion binding and oxidoreductase activity, whereas lipid synthesis and oxidative stress-related functions were suppressed.

Kyoto Encyclopedia of Genes and Genomes (KEGG) pathway analysis further supported these findings. The differentially expressed proteins in the ALI group were enriched primarily in pathways related to lipid metabolism and cell death, including fatty acid elongation, the PPAR signaling pathway, ferroptosis, apoptosis, chemical carcinogenesis-reactive oxygen species, alcoholic liver disease, the AMPK signaling pathway, and the HIF-1 signaling pathway (Fig. 7E). These findings confirmed the significant lipid peroxidation and deposition accompanying EtOH-ALI. Analysis of the differentially expressed proteins in the M2@SAM-F21 group (Fig. 7F) revealed the following mechanism of action: significant activation of immune-related pathways (*e.g.*, leukocyte *trans*-endothelial migration, nicotinate metabolism and nicotinamide metabolism) and significant suppression of pathways associated with lipid accumulation, oxidative stress, and cell death (*e.g.*, the PPAR signaling pathway, fatty acid metabolism, chemical carcinogenesis-reactive oxygen species, and ferroptosis). Collectively, these proteomic alterations, particularly the PPAR signaling pathway and HIF-1 signaling pathway, provide a molecular basis for the functional recovery observed in our models, namely the reduction in oxidative stress, suppression of apoptosis, and attenuation of inflammatory response.

Notably, as shown in Fig. 7E, M2@SAM-F21 treatment significantly reshaped core metabolic pathways associated with energy metabolism, including energy derivation by oxidation of organic compounds and ATP metabolic process. M2@SAM-F21 effectively blocked the polarization transition of macrophages from the resting state to the proinflammatory M1 phenotype—a process tightly coupled with metabolic reprogramming. The following key mechanisms are involved: 1) Metabolic switch regulation—the balance between glycolysis (proinflammatory) and oxidative phosphorylation (anti-inflammatory) directly determines the direction of macrophage polarization; and 2) oxidative stress promotion—ROS bursts accelerate inflammatory cascades by modifying metabolic enzyme activities. On the basis of these findings, we propose that M2@SAM-F21 reprograms the hepatic immune microenvironment by targeting macrophage metabolic reprogramming; specifically, by orchestrating energy metabolism remodeling and immune phenotype switching, M2@SAM-F21 simultaneously alleviates oxidative stress and cytokine storms, ultimately reestablishing metabolic–immune homeostasis to promote pathological resolution in ALI. This discovery reveals, for the first time, the therapeutic synergy of targeting the “metabolic–immune” dual axis in liver disease treatment. To further investigate the dynamic expression patterns and functional associations of differentially expressed proteins, we performed fuzzy clustering analysis of the consolidated differentially expressed proteins set, categorizing them into distinct clusters on the basis of expression profiles (Supporting Information Figs. S55–S65). Representative clusters 3, 5, and 7 were selected for GO and KEGG enrichment analyses. The expression of proteins in cluster 3 was significantly upregulated in the model group and partially restored posttreatment, whereas that of proteins in clusters 5 and 7 was specifically upregulated in the treatment group. GO/KEGG enrichment analyses revealed that these clusters were functionally enriched in immune response regulation (cluster 3), oxidative stress metabolism (cluster 5), and cell cycle control (cluster 7), indicating synergistic hepatoprotection by M2@SAM-F21 *via* coordinated multipathway regulation.

Furthermore, we utilized the STRING database to construct a protein‒protein interaction (PPI) network of the differentially expressed proteins. As shown in Fig. 7G, network analysis revealed the significant activation of core stress-metabolic pathways—alcohol/ROS metabolism, *β*-oxidation, and Golgi transport—in ALI. M2@SAM-F21 suppressed these pathways, demonstrating multitarget remediation of hepatic oxidative/lipid/metabolic pathology. In light of these findings, we validated our findings *via* Western blotting. The results (Fig. 7H) revealed that the expression of the key glycolytic enzymes HK2 and PKM2 was significantly elevated in the ALI group, whereas M2@SAM-F21 treatment effectively reversed this trend. This protein-level observation was further supported by concordant qPCR data (Supporting Information Fig. S66), demonstrating that M2@SAM-F21 modulates glycolysis at the transcriptional level. Similarly, changes in the expression of other key proteins (HO-1, SOD2, CD36, ACOX1) were also corroborated by their protein levels and mRNA profiles, yielding a consistent mechanistic picture across both analytical levels. Core to the efficacy of M2@SAM-F21 is its targeted modulation of metabolic reprogramming, which synergistically suppresses oxidative stress and proinflammatory signaling, culminating in the restoration of hepatic microenvironmental equilibrium for the treatment of ALI.

## 4 Discussion

The curative options for ALI, a major public health challenge with a complex pathogenesis, are limited beyond costly and scarce liver transplantation. Pathogenically, dysregulated oxidative stress and inflammatory cascades constitute core drivers of hepatic parenchymal damage across ALI subtypes^63^, ^64^, ^65^. In this study, we successfully constructed an M2 macrophage membrane-coated, diselenide-bonded mesoporous silica nano co-delivery system, M2@SAM-F21, providing a novel therapeutic solution for ALI. This system innovatively integrates three innovative strategies: 1) Biomimetic targeted delivery—specifically, the M2 macrophage membrane coating endows the system with precise inflammatory-targeting capability and prolonged circulation stability, significantly enhancing drug enrichment efficiency at the liver injury site; 2) pathological microenvironment-responsive drug release—the diselenide bonds enable intelligent, ROS-triggered drug release within the pathological microenvironment; and 3) synergistic gene‒drug codelivery. This multimechanism synergistic intervention platform achieves coordinated therapeutic enhancement through the combined action of exogenously administered silymarin and endogenously expressed FGF21 protein. The system efficiently scavenges ROS (O_2_·^−^, ·OH, and H_2_O_2_) in the liver injury microenvironment while concurrently activating the Nrf2/HO-1 signaling axis to restore immune homeostasis through the reversal of proinflammatory M1 macrophage polarization—significantly suppressing the release of inflammatory cytokines (TNF-*α* and IL-6) while enhancing the anti-inflammatory effects mediated by IL-10. Additionally, M2@SAM-F21 specifically inhibits key lipid-synthesizing enzymes to remodel metabolic networks, ultimately achieving the concerted suppression of oxidative stress storms, inflammatory cascade responses, and pathological lipid accumulation; furthermore, its unique “therapeutic-preventive integration” design expands the clinical intervention window, offering not only effective treatment for established liver injury but also potential prophylactic protective functions.

## 5 Conclusions

In summary, this study confirms the significant therapeutic potential of M2@SAM-F21 against ALI and highlights its promising potential as a nanotherapeutic candidate. Confirmation of its potential for “both symptomatic relief and root-cause treatment” across disease spectra would substantially increase its clinical value. This work achieves multidimensional innovations at the material level (biomimetic targeting carriers), biological level (multipathway synergistic mechanisms), and therapeutic level (integrated therapeutic and preventive applications), providing an innovative solution for ALI. This study not only vigorously propels the development of liver-targeted nanomedicines but also establishes a new paradigm for the precise intervention of oxidative stress-related diseases.

## Author contributions

Xinyu Tan: Writing – original draft, Visualization, Methodology, Investigation, Formal analysis, Data curation, Conceptualization. Man Li: Writing – review & editing, Writing – original draft, Visualization, Supervision, Methodology, Investigation. Zhibin Lan: Writing – review & editing, Conceptualization. Wenxiang Zhang: Visualization, Methodology, Investigation, Data curation. Junjie Zou: Methodology, Investigation, Formal analysis, Data curation. Yibo An & Wenjie Ni: Investigation, Data curation. Jingwen Zhen & Sheng Lin: Supervision, Investigation, Conceptualization. Xuwen Xiang & Jing Lin & Lin Che: Supervision, Conceptualization. Gang Liu: Writing – review & editing, Supervision, Resources. Jing Chen: Writing – review & editing, Supervision, Formal analysis, Funding acquisition, Conceptualization. Zhixiang Lu: Writing – review & editing, Supervision, Formal analysis, Project administration, Funding acquisition, Conceptualization.

## Conflicts of interest

The authors declare that they have no conflicts of interest.

## Appendix A Supporting information

The following is the Supporting Information to this article:
