# Supplementary material for: Dual-mode hepatoprotective nanoplatform combines ROS scavenging and FGF21 activation for precision acute liver injury therapy
Source: Acta Pharm Sin B. 2026 Mar 11;16(11):7744–65. doi: 10.1016/j.apsb.2026.03.005 (PMC13729529; doi:10.1016/j.apsb.2026.03.005)
Supplement: Supplementary file 1 [file mmc1.docx]

**Supporting Information for**

**Original article**

**Dual-mode hepatoprotective nanoplatform combines ROS scavenging and FGF21 activation for precision acute liver injury therapy**

**Xinyu Tan^a,b,f,†^, Man Li^b,f,†^, Zhibin Lan^d,†^,** **Wenxiang Zhang^b^, Junjie Zou^c^,** **Yibo An^b^, Jingwen Zheng^b^, Xuwen Xiang^c^,** **Jing Lin^b^, Sheng Lin^b^,** **Wenjie Ni^b^, Lin Che^e^, Gang Liu^c^, Jing Chen^a,*^, Zhixiang Lu^b,f,*^**

^a^*Department of Child Health, Department of Pediatrics, Women and Children’s Hospital, School of Life Sciences~~Medicine & School of Public Health~~, Xiamen University, Xiamen 361102, China*

^b^*State Key Laboratory of Vaccines for Infectious Diseases, Xiang An Biomedicine Laboratory, Fujian Provincial Key Laboratory of Innovative Drug Target Research, School of Pharmaceutical Sciences, Xiamen University, Xiamen 361102, China*

^c^*State Key Laboratory of Molecular Vaccinology and Molecular Diagnostics, State Key Laboratory of Vaccines for Infectious Diseases, Center for Molecular Imaging and Translational Medicine, Xiang An Biomedicine Laboratory, School of Public Health, Xiamen University, Xiamen 361102, China*

^d^*Fuzhou University Affiliated Provincial Hospital, School of Medicine,* *Fuzhou University, Fuzhou 350108, China*

^e^*State Key Laboratory of Oncology in South China, Sun Yat-sen University Cancer Center, Guangzhou National Laboratory, Guangzhou 510005, China*

^f^*Sichuan Research Institute of Xiamen University, Chengdu 610000, China*

Received in 15 October 2025; received in revised form 30 December 2025; accepted in 13 January 2026

^*^Corresponding authors.

E-mail addresses:chenjing8469899@126.com (Jing Chen), zhixiangl@xmu.edu.cn (Zhixiang Lu).

^†^These authors made equally contributions to this work.

**1. Supporting figures**

**Figure S1** Dynamic laser scattering (DLS) analysis of MSN.

**Figure S2** DLS analysis of AMSN.

**Figure S3** Zeta potential analysis of MSN, AMSN, Sil-AMSN, and M2@SAM-F21.

**Figure S4** (A) XPS survey of the M2@SAM-F21. Peaks corresponding to C, O, Si, and Se are clearly detected, confirming the elemental composition of the material. (B) XPS spectra of Se 3d in M2@SAM-F21.

**Figure S5** DLS analysis of Sil-AMSN.

**Figure S6** Concentration curve of silymarin.

**Figure S7** Agarose electrophoresis analysis of Sil-AMSN/pFGF21 complex. The first lane is the marker, the weight ratios of Sil-AMSN/pFGF21 are 0–50:1.

**Figure S8** DLS analysis of M2@SAM-F21.

**Figure S9** UV–Vis absorption spectra of MSN, AMSN, and M2@SAM-F21.

**Figure S10** Elemental mapping; BF represents bright-field images; P, Se, O, and Si represent different elemental fluorescent signals; and Merge is the overlapping image. Scale bar: 50 nm.

**Figure S11** Release profile of silymarin from M2@SAM-F21 under varied pH and oxidative conditions. Mean ± SD (*n* = 3).

**Figure S12** Transfection efficiency of M2@SAM‑F21 in AML12 cells. Flow cytometric quantification of GFP‑positive cells after treatment with the indicated formulations.

**Figure S13** Immunofluorescence images of cells treated with nanoparticles with or without M2 membrane coating. Blue fluorescence (DAPI) represents cell nuclei, green fluorescence (GFP-tag) indicates FGF21 expression. Scale bar: 20 μm.

**Figure S14** DPPH‧ scavenging ability of MSN. Mean ± SD (*n* = 5).

**Figure S15** ABTS‧⁺ scavenging ability of MSN. Mean ± SD (*n* = 5).

**F****igure S16** The cell viability of RAW 264.7 incubated with MSN, Sil-AMSN, or M2@SAM-F21 at different concentrations for 24 h. Means ± SD (*n* = 5).

**Figure S17** The cell viability of AML-12 incubated with MSN, Sil-AMSN, or M2@SAM-F21 at different concentrations for 24 h. Mean ± SD (*n* = 5).

**Figure S18** Flow cytometry analysis of the fluorescence intensity of M2@SAM-F21-ICG in cells at 4 h under APAP treat or not. The percentage of cells with M2@SAM-F21-ICG fluorescence is indicated.

**Figure S19** Statistical analysis of M2@SAM-F21-ICG levels in AML-12 cells at different time points. Mean ± SD (*n* = 3), *****P* < 0.0001, One-way ANOVA.

**Figure S20** (A) Cellular uptake efficiency of different nanoparticle formulations at 4 h under APAP treat. Blue fluorescence (DAPI) indicates cell nuclei, and red fluorescence originates from the loaded ICG. Scale bar: 20 μm. (B) Flow cytometry analysis of the fluorescence intensity of different nanoparticle formulations at 4 h under APAP treat.

**Figure S21** Representative confocal microscopy images showing AML‑12 cells after 4 h of co‑incubation with different formulations. M2 membranes were pre‑incubated with RS102895, a specific CCR2 inhibitor. Blue fluorescence (DAPI) indicates cell nuclei, and red fluorescence originates from the loaded ICG. Scale bar: 20 μm

**Figure S22** Statistical analysis of ROS levels in AML-12 cells under different treatment conditions. Mean ± SD (*n* = 3), *****P* < 0.0001, One-way ANOVA.

**Figure S23** Representative fluorescence images illustrating mitochondrial membrane potential (MMP) in AML-12 cells, assessed using the JC-1 probe. Red fluorescence indicates JC-1 aggregates in healthy mitochondria with high membrane potential, while green fluorescence represents JC-1 monomers in depolarized mitochondria with low membrane potential. Scale bar: 50 μm.

**Figure S24** Quantitative analysis of MMP, determined by measuring the ratio of red (JC-1 aggregates) to green (JC-1 monomers) fluorescence intensity. Mean ± SD (*n* = 3), **P* < 0.05, *****P* < 0.0001, One-way ANOVA.

**Figure S25** Representative fluorescence images of the viability of AML-12 cells under different treatment conditions. Green fluorescence indicates live cells (calcein-AM), whereas red fluorescence indicates dead cells (PI). Scale bar: 200 μm.

**Figure S26** Corresponding statistical analysis of AML-12 cells apoptosis after different treatments. Mean ± SD (*n* = 3), *****P* < 0.0001, One-way ANOVA.

**Figure S27** Statistical graphs showing the relative fluorescence intensity of CD86 in each group. Mean ± SD (*n* = 3), ****P* < 0.001 and *****P* < 0.0001, One-way ANOVA.

**Figure S28** Statistical graphs showing the relative fluorescence intensity of CD206 in each group. Mean ± SD (*n* = 3), *****P* < 0.0001 and ns indicates no significance, One-way ANOVA.

**Figure S29** Mean fluorescence intensity (MFI) of CD86 in RAW 264.7 under different treatment conditions. Mean ± SD (*n* = 3), *****P* < 0.0001 and ns indicates no significance, One-way ANOVA.

**Figure S30** MFI of CD206 in RAW 264.7 under different treatment conditions. Mean ± SD (*n* = 3), ***P* < 0.01 and *****P* < 0.0001, One-way ANOVA.

**Figure S31** Intracellular CD206 (A) and CD86 (B) levels in RAW 264.7 cells analyzed by flow cytometry.

**Figure S32** Evaluation of *in vivo* toxicity of M2@SAM-F21 to major organs (heart, liver, spleen, lung and kidney) at 1 day after intravenous administration. Scale bar: 50 μm.

**Figure S33** Blood parameters in normal mice (control group), and mice intravenously injected with M2@SAM-F21, 1 day after injection. (Mean ± SD, *n* = 5)

**Figure S34** Blood parameters in normal mice (control group), and mice intravenously injected with M2@SAM-F21, 14 days after injection. (Mean ± SD, *n* = 5)

**Figure S35** *In vitro* hemolysis test of M2@SAM-F21. (Mean ± SD, *n* = 3)

**Figure S36** Fluorescence intensity as observed *via* *ex vivo* fluorescence imaging of major organs. (Mean ± SD, *n* = 5)

**Figure S37** Representative HE whole-mount imaging of liver tissue from different groups under the ETOH-ALI mouse model.

**Figure S38** Western blot analysis of FGF21 expression levels in different liver tissues, *β*-actin was used as a loading control.

**Figure S39** Immunohistochemical staining (IHC) of FGF21 in liver tissues. Representative images showing FGF21 expression (brown) in different treatment groups. Scale bar: 100 μm.

**Figure S40** Representative TUNEL assay images of liver tissues in different groups. Blue fluorescence corresponds to DAPI‑stained nuclei; red fluorescence indicates TUNEL‑positive apoptotic cells. Scale bar: 200 μm.

**Figure S41** Representative DHE staining images of liver tissues in different groups. Nuclei were stained with DAPI (blue); superoxide was detected by DHE (red). Scale bar: 200 μm.

**Figure S42** Representative immunofluorescence staining (F4/80; CD86) images of liver tissues in different groups. Immunofluorescence of liver sections showing DAPI-stained nuclei (blue), F4/80 (macrophages, red), and CD86 (M1, green); merged images presented. Scale bar: 200 μm.

**Figure S43** Representative immunofluorescence staining (F4/80; CD206) images of liver tissues in different groups. Immunofluorescence of liver sections showing DAPI-stained nuclei (blue), F4/80 (macrophages, red), and CD206 (M2, green); merged images presented. Scale bar: 200 μm.

**Figure S44** Flow cytometry analysis of F4/80/CD86 (A) and F4/80/CD206 (B) in liver tissues from different groups.

**Figure S45** IHC of Nrf-2 (A) and HO-1 (B) in liver tissues. Representative images showing proteins expression (brown) in different treatment groups. Scale bar: 100 μm.

**Figure S46** Schematic diagram of the experimental design for the APAP-induced ALI mouse model and M2@SAM-F21 treatment protocol.

**Figure S47** Liver index from different groups under APAP-ALI mouse model. (Mean ± SD, *n* = 5, **** *P* < 0.0001, ns means no significance and One-way ANOVA.)

**Figure S48** Representative HE whole-mount imaging of liver tissue from different groups under the APAP-ALI mouse model.

**Figure S49** Serum levels of ALT in different groups during the experimental period. (Mean ± SD, *n* = 5, ****P* < 0.001, **** *P* < 0.0001 and One-way ANOVA.)

**Figure S50** Serum levels of AST in different groups during the experimental period. (Mean ± SD, *n* = 5, ***P* < 0.01, *****P* < 0.0001 and One-way ANOVA.)

**Figure S51** Gene Ontology (GO) analysis of the up-regulated and down-regulated proteins in “Molecular Function” (MF) among Control-ALI comparison.

**Figure S52** GO analysis of the up-regulated and down-regulated proteins in “Cellular Component” (CC) among Control-ALI comparison.

**Figure S53** GO analysis of the up-regulated and down-regulated proteins in MF among ALI-M2@SAM-F21 comparison.

**Figure S54** GO analysis of the up-regulated and down-regulated proteins in “CC” among ALI-M2@SAM-F21 comparison.

**Figure S55** The trend analysis of proteomics in different groups.

**Figure S56** Kyoto Encyclopedia of Genes and Genomes (KEGG) pathway analysis (left) and GO analysis (right) of the differentially expressed proteins in mfuzz_3.

**Figure S57** KEGG pathway analysis (left) and GO analysis (right) of the differentially expressed proteins in mfuzz_5.

**Figure S58** KEGG pathway analysis (left) and GO analysis (right) of the differentially expressed proteins in mfuzz_7.

**Figure S59** A heatmap illustrating the expression patterns of differentially expressed proteins (DEPs) across various samples in HIF-1 signaling pathway.

**Figure S60** A heatmap illustrating the expression patterns of DEPs s across various samples in Fatty acid elongation.

**Figure S61** A heatmap illustrating the expression patterns of DEPs across various samples in Fatty acid metabolism.

**Figure S62** A heatmap illustrating the expression patterns of DEPs across various samples in Ferroptosis.

**Figure S63** A heatmap illustrating the expression patterns of DEPs across various samples in Chemical carcinogenesis-reactive oxygen species.

**Figure S64** A heatmap illustrating the expression patterns of DEPs across various samples in Alcoholic liver disease.

**Figure S65** A heatmap illustrating the expression patterns of DEPs across various samples in Phagosome.

**Figure S66** qPCR analysis showing the mRNA expression levels of *HO-1* (A), *SOD2* (B), *CD36* (C), *ACOX1* (D), *HK2* (E) and *PKM2* (F) in liver tissues. Mean ± SD, *n* = 5, ***P* < 0.01, ****P* < 0.001 and *****P* < 0.0001; one-way ANOVA.

**2. Supporting table**

**Table 1** Primers for qPCR.

| Primer | Forward | Reverse |
| --- | --- | --- |
| Mouse *Ho-1* | CACCGAGGCCACCAAGCGCTACGTG | AAACCACGTAGCGCTTGGTGGCCTC |
| Mouse *Sod2* | GTAGGGCCTGTCCGATGATG | CGCTACTGAGAAAGGTGCCA |
| Mouse *Cd36* | GCACCACTGTGTACAGACAG | GTGCAGCTGCTACAGCCAG |
| Mouse *Acox1* | UCAUCUAAGAGACCUAGGCTT | GCCUAGGUCUCUUAGAUGATT |
| Mouse *Hk2* | CAAGAAGCTCCCACTGGGTT | CAACGTCTCTGCCTTCCACT |
| Mouse *Pkm2* | GTCTGGAGAAACAGCCAAGG | CGGAGTTCCTCGAATAGCTG |
| Mouse *β-*actin | GTCGTACCACAGGCATTGTGATGG | GCAATGCCTGGGTACATGGTGG |
